# Solvent-Assisted Modification of Laser-Induced Graphene for Surface-Enhanced Electrochemical Response

**DOI:** 10.1021/acs.analchem.5c06456

**Published:** 2026-01-20

**Authors:** Nélio I. G. Inoque, Raquel G. Rocha, Gilvana P. Siqueira, Ana Clara Maia Oliveira, Michele V. C. O. Da Silva, Robert D. Crapnell, Craig E. Banks, Edson Nossol, Eduardo Mathias Richter, Rodrigo A. A. Muñoz

**Affiliations:** † Institute of Chemistry, 28119 Federal University of Uberlândia, Uberlândia 38400-902, Minas Gerais, Brazil; ‡ Faculty of Science and Engineering, 5289 Manchester Metropolitan University, Dalton Building, Chester Street, Manchester M1 5GD, Great Britain

## Abstract

The fabrication and application of laser-induced graphene (LIG) have received significant interest across various research fields, particularly in sensing technologies. Herein, we investigated the surface modification of LIG electrodes with different solvents and demonstrated that dimethyl sulfoxide (DMSO) enhances their electrochemical activity. Importantly, our findings reveal that solvent treatments themselves can induce significant modifications on the electrode surface (e.g., changes in functional groups, wettability, and morphology), which must be carefully considered in sensor design and optimization. The application of a microliter aliquot of DMSO to the LIG surface significantly altered its wettability, promoting a transition to hydrophilic behavior, which was verified by contact-angle measurements. AFM and XRD results indicate that DMSO treatment promotes the rearrangement of disordered carbon atoms, minimizing localized structural defects and enabling more efficient π–π stacking between graphene sheets. The cyclic voltammetric response of the [Fe­(CN)_6_]^3‑/4‑^ redox probe showed a 3-fold increase in peak current. The enhanced electrochemical effect of DMSO surface changes on LIG electrodes was also confirmed in the presence of several organic species, and a substantial current increase was verified by the effect of DMSO. As proof-of-concept, the antibiotic sulfanilamide was detected in synthetic urine and water samples using differential-pulse voltammetry on DMSO-treated LIG. Under optimized conditions, the sensor exhibited a linear response in the range of 0.3–9.0 μmol L^–1^ with a limit of detection of 0.09 μmol L^–1^ and satisfactory recovery values (90–104%).

## Graphical abstract

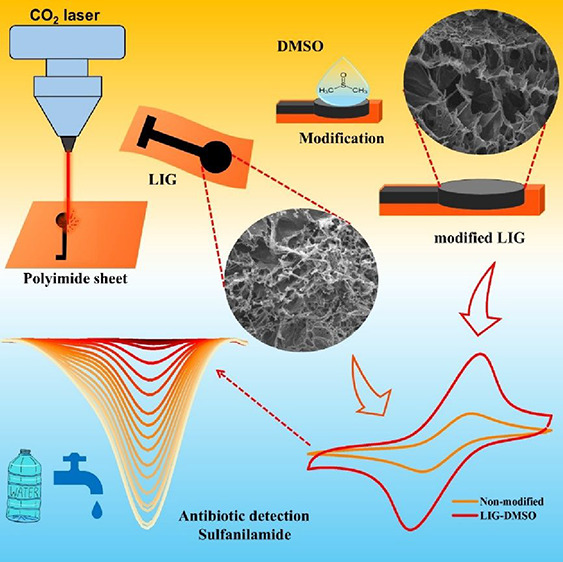

## Introduction

Graphene and its derivatives have been extensively explored in scientific research due to their great physical and chemical properties, such as a remarkably high surface area (∼2630 m^2^·g^–1^), mechanical strength, superior thermal conductivity (∼3000 W·m^–1^·K^–1^), high electrical conductivity (5–25 S cm^–1^), and biocompatibility.^1^ Its excellent electronic properties are linked to its monatomic layered structure, which is arranged in a honeycomb-like lattice.^2,3^ These characteristics make graphene highly attractive for the development of electrochemical devices, particularly in sensing applications.^2,4^ However, the practical implementation of graphene-based technologies remains limited by the need for simple, cost-effective, and low-temperature fabrication methods capable of producing high-quality graphene nanomaterials on a large scale.^5^

To address this challenge, laser scribing techniques have been widely explored in recent years for the large-scale fabrication of low-cost, reproducible devices suitable for laboratory environments.^4,6^ Electrochemical sensors based on graphene-like materials have been successfully developed using various laser sources, including CO_2_,^7,8^ UV,^9^ and blue lasers,^10^ being applied to different polymer surfaces.^11,12^ This laser-irradiation process relies on the photothermal conversion of sp^3^-hybridized carbon atoms in polymer substrates into sp^2^-hybridized carbon, under ambient conditions.^3,12^ In brief, carbon-based precursors absorb energy, causing vibrations in the crystalline lattice. These vibrations generate localized temperatures as high as ∼2500 °C, which break covalent bonds, particularly C–O and C–N, and lead to the release of gaseous byproducts. The remaining carbon atoms reorganize, resulting in the formation of the characteristic three-dimensional (3D) porous graphene-like structures known as laser-induced graphene (LIG).^13,14^

While LIG-based devices are capable of operating without prior surface modification,^15,16^ improvements in electrochemical performance are commonly achieved through postscribing surface treatments tailored to specific analytical applications.^17–19^

For instance, the surface of LIG electrodes fabricated from phenolic paper was electrochemically treated in a 1.0 mol·L^–1^ KCl solution by applying a potential of −1.0 V (*vs.* Ag) for 60 s to provide excellent conductivity and low charge-transfer resistance.^20^ LIG electrodes produced from phenolic resin substrates were also modified by drop-casting 5 μL of a functionalized multiwalled carbon nanotube (MWCNT) suspension onto the surface to enhance electrode conductivity and thus improve their electrochemical activity.^18^ Matias and coauthors demonstrated the applicability of electrochemically modified LIG surfaces with Prussian blue for the detection of hydrogen peroxide.^21^ Other studies have employed metal nanoparticles, such as Au,^22^ NiFe_2_O_4_ and CeO_2_ ^23^ nanoparticles, a mixture of Au, NiO, and Rh nanoparticles,^24^ Ag-based materials^19,25,26^ to enhance the electrochemical performance of LIG electrodes for sensing applications Although these surface treatments have demonstrated improvements in the electrochemical response of LIG electrodes, they typically require multiple steps, additional reagents or nanomaterials, and more complex preparation procedures, which increase cost and limit scalability, and their performance still offers room for further enhancement.

Zhang’s group demonstrated that the addition of ethanol to the surface of LIG resulted in modifications to its morphology and composition, leading to a transition from pinning to rolling.^27,28^ The authors specifically highlighted the superhydrophobic surface transition and utilized this material for Raman scattering detection. However, to the best of our knowledge, no studies have investigated the effects of solvent treatments on the electrochemical activity of the LIG surfaces. Therefore, there is a clear need to explore how solvents influence graphene-based nanostructures, which could pave the way for the development of a wide range of electrochemical devices.

In this paper, we demonstrate that a simple solvent treatment using dimethyl sulfoxide (DMSO) enhances the electrochemical activity of LIG electrodes due to changes in both surface chemistry and surface morphology. For this investigation, LIG electrodes were fabricated from polyimide (PI) sheets using a CO_2_ laser source, and voltammetric measurements of various electroactive species, such as chloramphenicol, sulfanilamide (SFL), ascorbic acid, amoxicillin, ciprofloxacin, and tetracycline, were assessed. These analytes were selected due to their relevance in the food industry and/or their potential occurrence in wastewater,^29–31^ providing a practical framework to evaluate the performance of the modified electrodes for compounds of industrial and environmental significance.

To confirm long-term surface changes, the DMSO-treated LIG electrodes were evaluated for SFL detection in real-world water samples. We believe this simple and effective approach represents a promising route for the large-scale production of high-quality graphene-like structures for electrochemical applications.

## Experimental Section

See more in the Supporting Information

### Production of LIG Electrodes

A CO_2_ laser cutting and engraving machine (10.6 μm, 40 W) from WorkSpecial WS4040 (São Paulo, Brazil) was used to engrave the polyimide substrate with a layer thickness of 0.15 mm (Vemar, Sorocaba, Brazil) for electrode fabrication. The working electrode design was created using RDWorks 8.0 software. The steps involved in the production of LIG are summarized in Figure 1.

**Figure 1:**
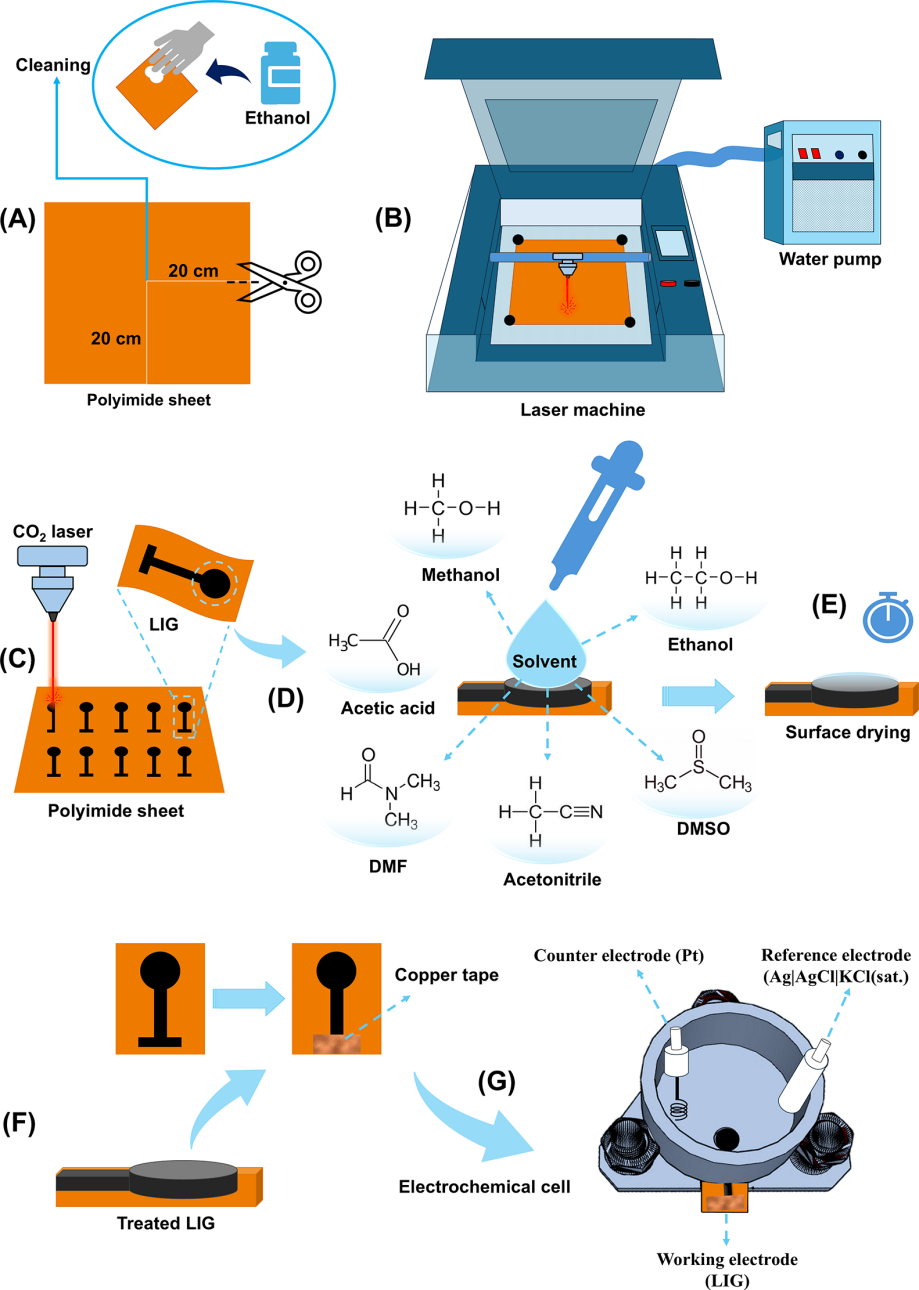
Schematic representation of the preparation, fabrication, and modification of the LIG electrodes, as well as their integration into the electrochemical cell. (A) Cutting and cleaning of the polyimide sheet; (B) positioning of the polyimide sheet in the CO_2_ laser machine; (C) highlighting the laser engraving process of the electrodes; (D) drop-casting modification, in which 3 μL of solvent was applied to the LIG surface; (E) drying the electrodes at room temperature; (F) attaching copper tape to the LIG electrodes to secure the potentiostat connectors; (G) the modified LIG electrode is placed at the bottom of the 3D-printed electrochemical cell with platinum counter and Ag|AgCl|KCl_(sat.)_ reference electrodes inserted through the top of the cell.

Initially, the polyimide sheet was cleaned by using tissue paper and ethanol. The laser machine, coupled with a water pump for temperature control of the laser source, was then used to engrave the polyimide sheet, which was fixed to the cutting table using magnets (Figure 1B). The engraving was carried out at a power of 1.0 W (measured by a power meter), a speed of 40 mm s^–1^, a scan gap of 0.1 mm, and a working distance of 10 mm between the substrate and laser head. The fabrication of 10 electrodes was completed in 5.0 min (Figure 1C). These parameters were previously optimized by Sevene et al.^32^

In the solvent treatment step, LIG electrodes were modified by drop-casting 3.0 μL of solvent, acetonitrile (ACN), dimethylformamide (DMF), ethanol (EtOH), methanol (MeOH), acetic acid (HAc), or DMSO, directly onto the electrode surface (Figure 1D). This volume was previously optimized to ensure complete and uniform coverage of the working electrode without an overflow. Smaller volumes failed to fully wet the surface, leading to inconsistent contact, while larger volumes exceeded the electrode boundaries, potentially interfering with the measurements. Therefore, 3.0 μL was established as the optimal volume for reliable and reproducible deposition. After drop-casting, the electrodes were washed with deionized water and dried at room temperature (∼25 °C) for 5 h (Figure 1E).

After this time, the electrodes were washed with deionized water several times. A duration optimized based on intraelectrode precision measurements using the [Fe­(CN)_6_]^3‑/4‑^ redox probe to ensure stable and reproducible surface modification (data not shown).

Prior to use, copper tape was applied to the electrical contact of the LIG electrodes to facilitate the attachment of the potentiostat connectors (Figure 1F). The modified electrodes were then inserted into the lower compartment of the 3D-printed electrochemical cell for electrochemical measurements, as shown in Figure 1G.

## Results and Discussion

### Preliminary Electrochemical Investigation of LIG as a Function of Surface Modification with Different Solvents

To investigate the effect of different solvents, including DMSO, ACN, EtOH, MeOH, HAc, and DMF, on the electrochemical response of LIG, CV measurements were performed using 1.0 mmol L^–1^ [Fe­(CN)_6_]^3–/4–^ redox probe in 0.10 mol L^–1^ KCl solution at 50 mV s^–1^. LIG electrodes modified with each solvent, as described in the Experimental section, were evaluated under the same conditions (Figure 2). As observed, the electrochemical profiles revealed a significant increase in the current response for electrodes treated with DMF (∼1.3-fold), HAc (∼1.6-fold), and DMSO (∼2.7-fold). In contrast, modifications with ACN and EtOH did not lead to a notable enhancement in the anodic or cathodic peak currents. Interestingly, MeOH treatment resulted in a decrease in current intensity, with peak currents reduced by approximately 0.67-fold. Based on the superior electrochemical performance, DMSO was selected as the optimal solvent for subsequent experiments.

**Figure 2:**
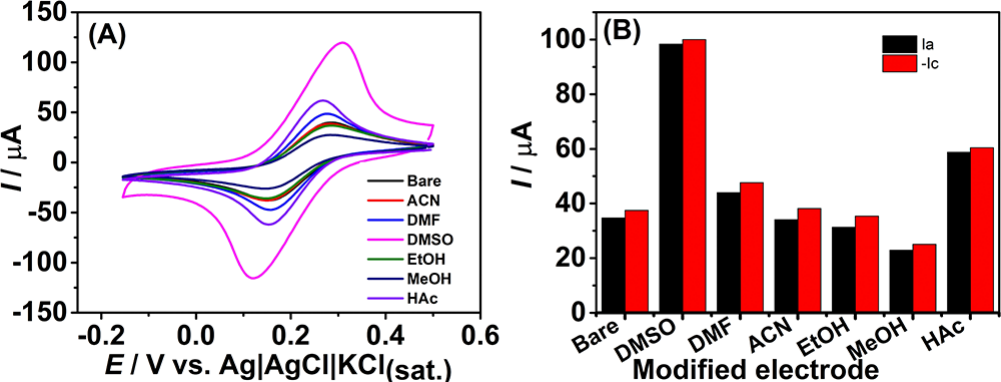
(A) CVs recorded in the presence of 1.0 mmol L^–1^ [Fe­(CN)_6_]^3‑/4‑^ in 0.10 mol L^–1^ KCl solution, using unmodified and solvent-modified electrodes; (B) anodic (*I*_a_, black bar) and cathodic (−*I*_c_, red bar) currents extracted from the data shown in Figure 2A. CV conditions: step potential = 5 mV; scan rate: 50 mV s^–1^.

CV experiments were carried out to evaluate the influence of the DMSO drop-cast volume on the electrochemical performance of the LIG electrodes. The volume was varied from 1 to 7 μL on a working electrode area of 0.19 cm^2^, and the current response was monitored accordingly. As shown in Figure S1, the current increased progressively up to 3 μL, reaching the maximum enhancement under this condition. For volumes >3 μL, the current response remained nearly constant, indicating that additional solvent loading did not further improve the electrode performance. Therefore, 3 μL was selected as the optimal volume for subsequent experiments, ensuring reproducibility and efficient sensor performance.

Long-term stability of the DMSO-modified LIG electrodes was evaluated through successive cyclic voltammetry measurements (*n* = 100) in the presence of 1.0 mmol L^–1^ [Fe­(CN)_6_]^3‑/4‑^, Figure S2. The relative standard deviation (RSD) for both anodic and cathodic currents was below 1.72%, indicating that the surface modification remains stable over time.

Next, morphological and spectroscopic characterizations (XPS, Raman spectroscopy, SEM imaging, and contact angle measurements) were performed to investigate the structural and surface changes that may have contributed to the observed current enhancement.

### Spectroscopic and Morphological Characterization of Bare and Modified LIG Electrodes

Initially, the LIG electrode surfaces were analyzed using SEM images (Figures 3 and S3). Both surfaces (before and after solvent treatment) exhibited a similar porous morphology, with pore sizes ranging from 2 to 8 μm, consisting of a combination of sheet-like and strip-like graphene structures. As widely reported in the literature, this porous graphene-like morphology results from the high localized temperatures (>2500 °C) generated by laser irradiation in the ablated region.^3,33^ These conditions promote the carbonization of the organic substrate and the release of gaseous byproducts, ultimately leading to the formation of the observed porous architecture.^20^

**Figure 3:**
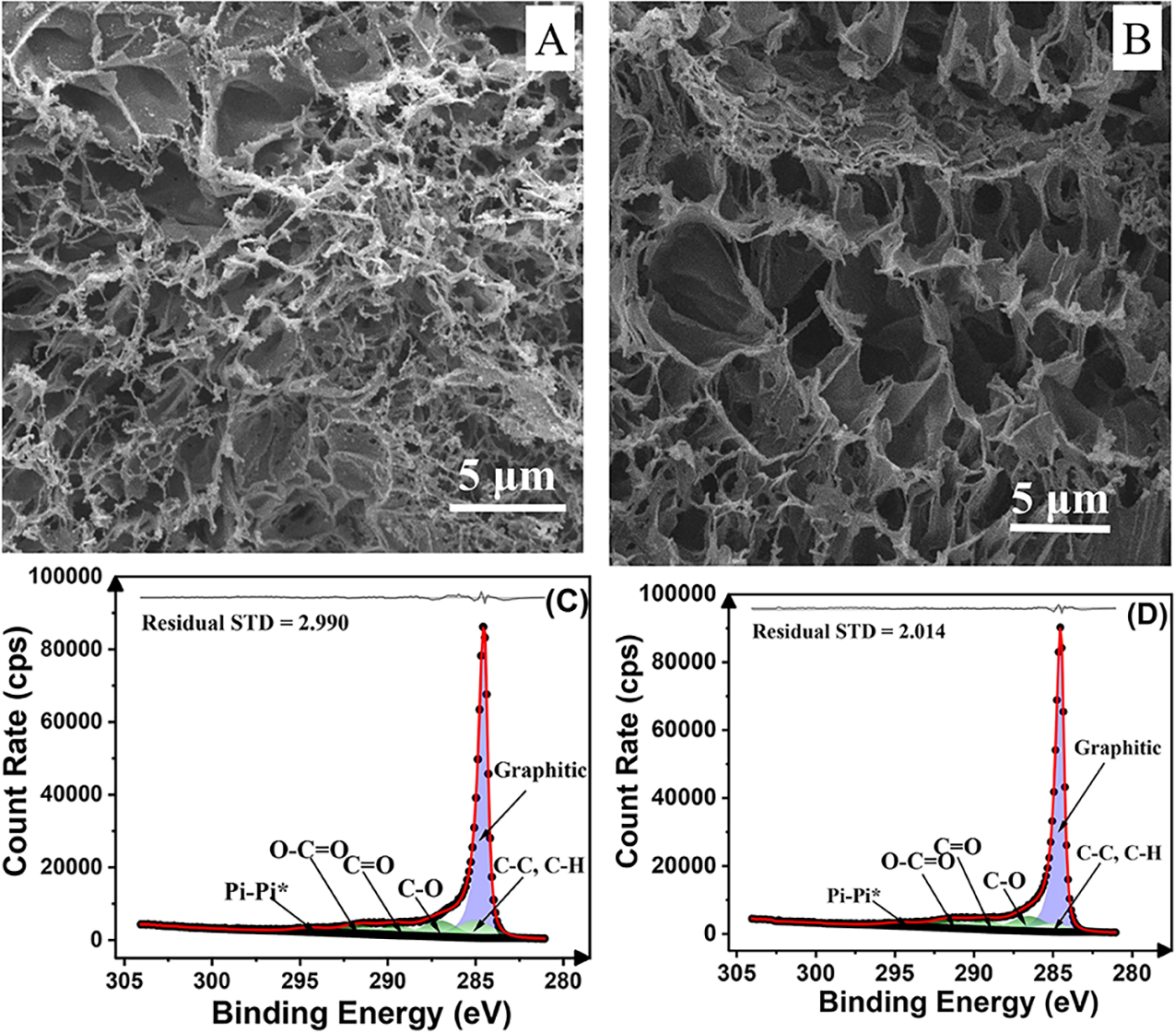
SEM images of the (A) bare LIG electrode and (B) DMSO-treated LIG electrode surfaces. XPS survey spectra and high-resolution deconvoluted C 1s components were obtained for (C) bare and (D) DMSO-treated LIG electrodes, respectively.

Despite the overall similarity in surface morphology between bare and DMSO-treated LIG, the treated electrodes exhibited a more dendritic texture with an increased number of surface capillaries. This microstructural feature may facilitate water infiltration into the fiber network, promoting droplet pinning on the LIG surface and enhancing the hydrophilicity of the electrode surface.^28^

In addition, morphological surface analysis revealed significant differences between the untreated LIG electrode and those subjected to DMSO and ethanol treatments, as shown in Figure S4. After treatment, the electrodes exhibited a more dendritic surface texture, with an increased number and distribution of capillaries within the microstructure. This more branched morphology facilitates solution infiltration into the graphene fiber network, enhancing droplet retention on the surface and consequently increasing the material’s hydrophilicity, an essential characteristic for electrochemical applications in aqueous media.^34^ The more pronounced increase in dendritic texture observed in the DMSO-treated LIG electrode compared to the ethanol treatment can be attributed to the polar and aprotic nature of this solvent. DMSO has a high dielectric constant and strong solvation capability, which allows it to interact intensely with functional groups present on the LIG surface. This interaction may promote surface reorientation or reorganization of graphene domains and functional residues, contributing to the formation of more branched and porous structures.

Additionally, DMSO may facilitate the removal of residual organic compounds left behind from the laser processing, further exposing the porous structure and favoring the development of channels and surface roughness.^27,34^ The EDS spectra of the LIG electrodes in Figure S5 confirmed predominant carbon (consistent with the graphitic carbon network) and detectable oxygen (from surface oxygen-containing groups).

Notably, sulfur was present exclusively in the DMSO-treated samples, suggesting either residual adsorption or surface incorporation of sulfur-containing moieties from DMSO. This observation aligns with previous findings that DMSO can act as a sulfur source and interact strongly with carbon-based surfaces, leading to structural modifications and sulfur retention.^35^ The Au signal in all spectra corresponds to the thin gold layer sputter-deposited prior to SEM/EDS imaging, applied to prevent surface charging effects and material degradation under the electron beam.

Next, water contact angle measurements were analyzed to understand whether these solvent modifications altered the wettability properties of the LIG surface microstructure. The unmodified LIG electrode showed a contact angle of 106°, due to the hydrophobic nature of the surface, which is basically composed of carbon, as shown in Figure S6A. However, the surface of LIG after treatment with EtOH (Figure S6B), MeOH (Figure S6C), and ACN (Figure S6D) exhibited greater hydrophobicity compared to the unmodified LIG, with contact angle values ranging from 108° to 111°. These findings indicated that the water adhesion slightly decreased after EtOH, MeOH, and ACN treatment, resulting in a transition from pinning to rolling. These results agreed with those observed by Han and co-workers,^27^ who showed a superhydrophobic LIG surface after ethanol treatment.

On the other hand, a different effect was observed after modification of LIG with solvents such as DMF (Figure S6E) and DMSO (Figure S6F). LIG treated with DMF increased its wettability, decreasing the contact angle to 74°. A highly wettable surface was observed after LIG treatment with DMSO, resulting in a contact angle of 32°. These solvents promoted an increase in porosity in the graphene fibers on the surface of the capillary-shaped LIG electrodes (as observed in the SEM images, Figures 3B and S3B), facilitating capillary attraction of water into the fibers and the adhesion of droplets to the LIG surface, thereby reducing the contact angles. The presence of carboxyl groups induces microhydrophilic areas on the LIG surface; however, after treatment with ethanol, methanol, and acetonitrile, these areas are weakened or eliminated, resulting in a more hydrophobic surface.^27,36,37^

In fact, as shown in Figure 2, Cassidy et al.^38^ evaluated how the hydrophilicity of different surfaces affects the electrochemical activity of [Fe­(CN)_6_]^3‑/4‑^, and observed that more hydrophilic surfaces promote faster heterogeneous electron transfer (HET) and higher current intensities.

Carbon-based materials, such as graphene, are typically identified and characterized using Raman spectroscopy. The Raman spectra obtained for untreated and DMSO-treated LIG surfaces (Figure S7) revealed three characteristic bands associated with graphene-based materials: the D band (1366 cm^–1^), related to structural defects or disorder in the material; the G band (1591 cm^–1^), corresponding to the in-plane vibration of sp^2^-hybridized carbon atoms; and the 2D band (2697 cm^–1^), indicative of the number of stacked graphene layers present in the material.^3,39,40^ In addition to these main features, other peaks were also observed and are attributed to structural defects in the graphene, mainly caused by the presence of heteroatoms such as nitrogen and oxygen originating from the polyimide precursor.^41^ The Raman spectra of the LIG electrode strongly suggest the formation of a graphene-like material, providing clear evidence of its structural features. Since LIG is a graphene-based material characterized by a high density of defects,^6,42^ the *I*_D_/*I*_G_ ratio is commonly used to estimate the degree of graphitization or crystallinity within the porous structure. This ratio increases with the level of disorder present in the graphene lattice. The *I*_D_/*I*_G_ ratio for the untreated LIG surface was 0.84, while the DMSO-treated LIG surface showed a slightly higher value of 0.91. These results suggest that minor structural defects were introduced onto the graphene surface as a result of the DMSO treatment, probably due to the insertion of carbon–oxygen functional groups.^43^

XPS analyses were performed to further investigate the chemical composition of both untreated and DMSO-treated LIG electrodes. Figure 3C,D shows the high-resolution C 1s spectra for the bare LIG and DMSO-treated LIG electrodes, respectively, while the corresponding survey scans and O 1s spectra are provided in Figure S8. The survey scans (Figure S8) confirm the presence of carbon and oxygen in both samples, consistent with the expected composition of graphene-based materials. The deconvolution of the C 1s spectra required four symmetric peaks for accurate fitting, along with a dominant asymmetric peak centered at 284.5 eV, attributed to graphitic (sp^2^) carbon,^44^ indicating that the LIG structure is primarily composed of sp^2^-hybridized carbon atoms.^45^ Additionally, a peak associated with sp^3^-hybridized C–C bonding was observed near 285.0 eV, along with smaller contributions from C=O, O–C=O, and π–π* interactions, characteristic of the surface chemistry of LIG.

A comparison of the C 1s spectra (Figure 3) revealed an increase in the intensity of the C=O peaks in the DMSO-treated electrode. The increase in oxygen-containing functional groups is likely a result of the interaction of DMSO with the graphene surface, leading to surface oxidation and functionalization. Quantitative analysis confirmed this trend, showing an increase in the atomic concentration of C=O from 5.72% to 6.96% after DMSO treatment (Table S1).

According to the literature,^27,46,47^ C–OH and C=O functional groups tend to interact readily with water molecules, promoting hydrophilic properties. In contrast, C–O–C and C–C groups exhibit limited interactions with water, thus contributing to hydrophobic behavior. In this study, the DMSO-treated LIG surface contained a significant amount of C–O and C=O groups along with a three-dimensional (3D) porous structure composed of microfibers and defects. This morphology resulted in a lower contact angle, indicating the presence of microhydrophilic regions with strong affinity for water, as observed by other results described above.

Surface area analyses obtained from BET isotherms (Figure 4) revealed that the ethanol-treated LIG electrode exhibited the highest specific surface area (1193.5 m^2^/g), followed by the DMSO-treated (999.30 m^2^/g) and the unmodified electrode (921.065 m^2^/g). This increase suggests that ethanol enhances porosity and surface irregularities, whereas DMSO induces structural reorganization that improves electrical conductivity without substantially enlarging the surface area. Therefore, both the surface area and structural integrity play critical roles in determining the electrochemical performance of the electrodes.

**Figure 4:**
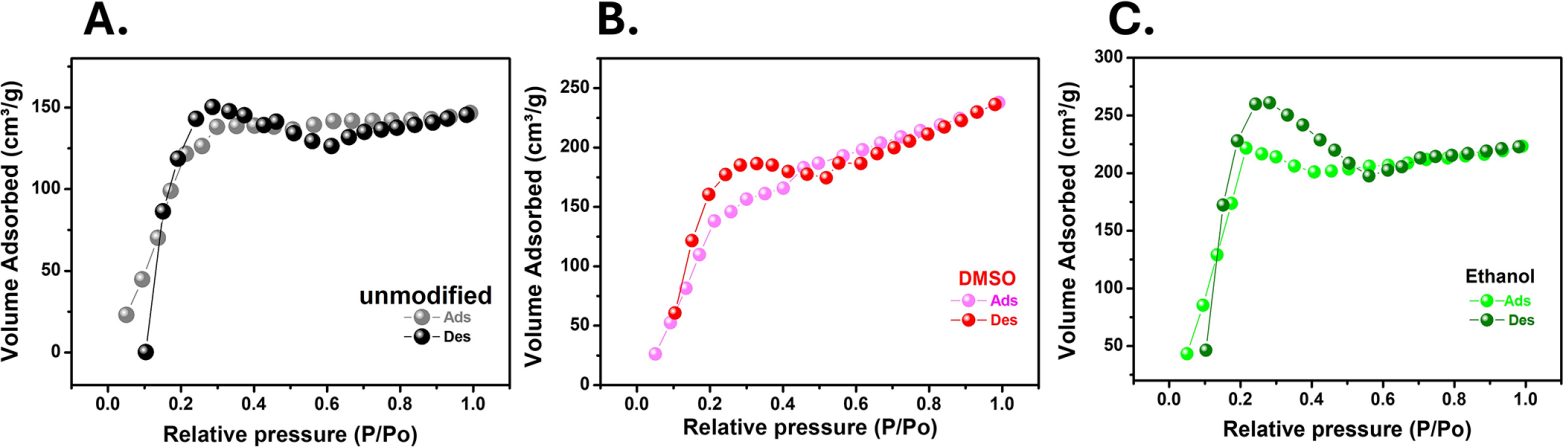
Nitrogen (N_2_) adsorption–desorption isotherms obtained by the BET method for LIG electrodes: (A) unmodified LIG, (B) LIG treated with DMSO, and (C) LIG treated with ethanol.

LIG electrodes were analyzed by AFM, as shown in Figure 5. This characterization enabled the evaluation of roughness and morphology parameters, which are fundamental for understanding the material’s properties. The calculated average roughness (*S*_a_) values were 266.2, 429.5, and 316.8 nm for the unmodified LIG, LIG/DMSO, and LIG/EtOH, respectively. Although the DMSO treatment did not lead to a significant increase in surface area, a substantial rise in roughness was observed, which is associated with its high polarity and strong interaction with carbonaceous structures. This effect suggests a structural reorganization within the LIG, capable of reducing defects and promoting the formation of more graphitic domains, thereby enhancing electrical conductivity. Thus, while ethanol primarily contributes to increasing the surface area, DMSO stands out for improving structural quality, justifying its use as a modifier to optimize the electrochemical performance of the electrodes.^27,34^

**Figure 5:**
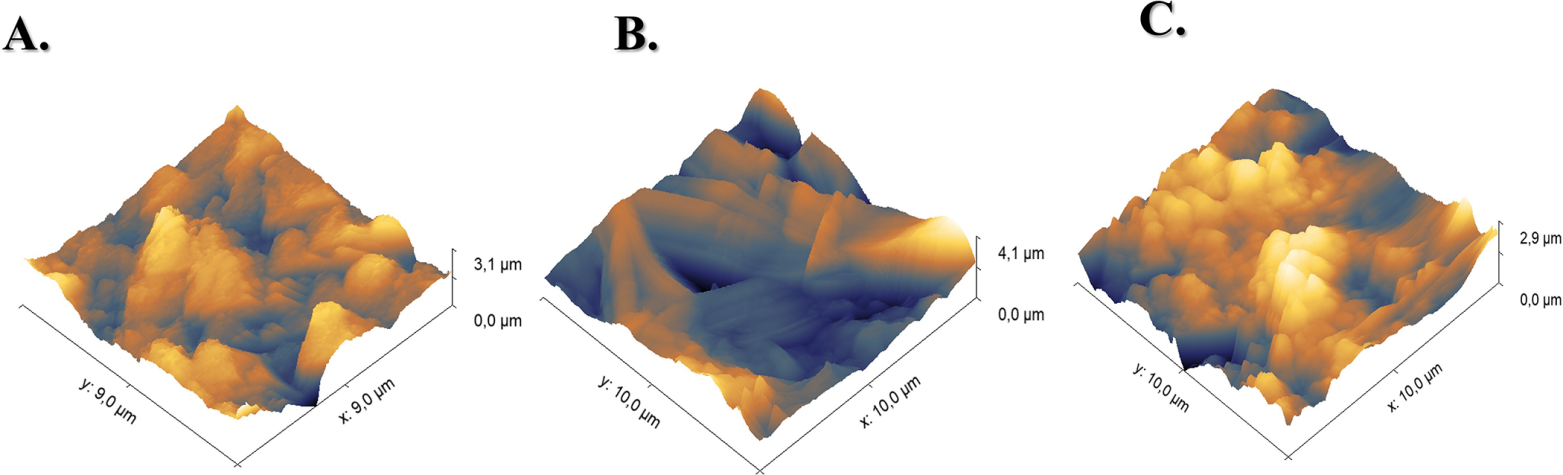
3D AFM surface images of LIG electrodes: (A) unmodified LIG (*S*_a_ = 266.2 nm), (B) LIG/DMSO (*S*_a_ = 429.5 nm), and (C) LIG/EtOH (*S*_a_ = 316.8 nm).

These findings were further supported by XRD results, in which unmodified and ethanol-treated LIG displayed diffraction peaks at 26°, 21°, and 14°, whereas the DMSO-treated electrode exhibited only the peaks at 26° and 21°. The peak at 26° corresponds to the (002) plane of graphite, characteristic of ordered graphitic domains; the peak at 21° is associated with partially ordered or distorted carbon structures; and the peak at 14° is related to larger interplanar spacing, typically found in defective or functionalized regions. The absence of the 14° peak in the DMSO-treated sample indicates that the solvent-induced reorganization and compaction of the graphene layers occurred, reducing defects and enhancing structural order.^48^

In contrast, ethanol preserved some of these structural irregularities, as evidenced by the persistence of the 14° peak and the higher surface area. This behavior has been attributed to ethanol’s ability to assist in repairing defects in oxidized graphene through the formation of new hexagonal bonds, while still maintaining expanded structural features. Altogether, these results demonstrate that DMSO acts as a structural ordering agent, improving the graphitic domains and the conductivity. As illustrated in Figure 6, the XRD patterns of the unmodified LIG, LIG/DMSO, and LIG/EtOH samples reveal distinct structural features.

**Figure 6:**
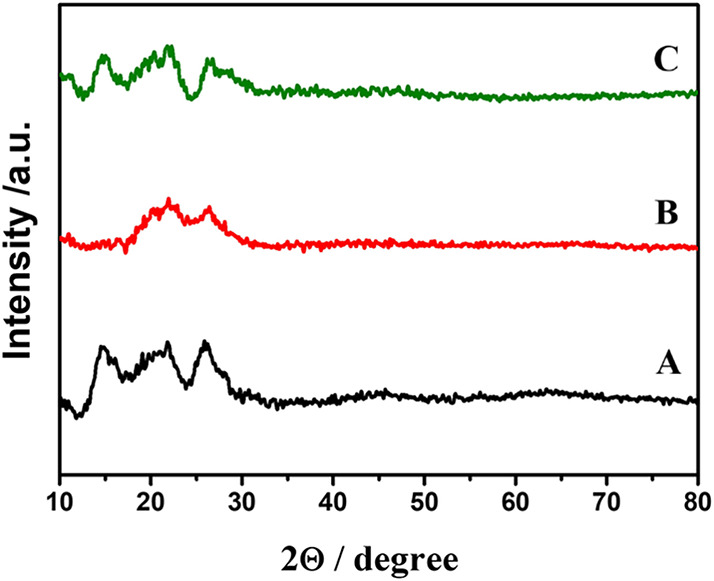
XRD patterns of laser-induced graphene (LIG) electrodes: (A) unmodified, (B) DMSO-treated, and (C) ethanol-treated.

AFM and XRD results indicate that DMSO treatment promotes the rearrangement of disordered carbon atoms, minimizing localized structural defects and enabling a more efficient π–π stacking between graphene sheets. This enhanced structural ordering reduces electron scattering at defective sites, resulting in improved charge transport, as further supported by the EIS data.^49^ Figure S9 shows a schematic illustration of some modifications that can occur on the electrode LIG surface after DMSO-assisted treatment.

### Electrochemical Characterization of DMSO-Treated LIG Electrodes

As previously shown in Figure 2, surface modification of LIG with DMSO provided a 2.8-fold increase in the anodic and cathodic peak currents in the cyclic voltammograms for 1.0 mmol L^–1^ of [Fe­(CN)_6_]^3–/4–^. Subsequently, a scan rate study was performed in the range of 10–100 mV s^–1^ for both untreated and DMSO-treated electrodes. A linear relationship between peak current and the square root of the scan rate was observed, with the slope increasing by approximately 2.1 times after DMSO treatment (Figure S10). These results indicate that a diffusion-controlled process governs the electrochemical response of the ferricyanide probe on both electrode surfaces.

Given the significant differences in the electrochemical responses observed between untreated and treated electrodes, we investigated whether these changes could be attributed to variations in the electroactive area (*A*_e__l__e_). Some works have estimated the *A*_ele_ using the Randles–Sevick equation for reversible systems, using CV scan rate experiments, according to eq 1.^50^

$$I_{p}=\pm 0.446\;{nFCA}_{\mathrm{ele}}\sqrt{\frac{nFD\nu }{RT}}$$ 1

In this equation, *R* is the constant gas, *n* is the number of electrons, *F* is Faraday’s constant, *C is* the concentration, *D* is the diffusion coefficient, and *T* is the temperature. Therefore, based on CV experiments conducted in the presence of 1.0 mmol L^–1^ [Fe­(CN)_6_]^3‑/4‑^ in 0.10 mol L^–1^ KCl solution at different scan rates (Figure S10), *A*_ele_ bare and DMSO-treated LIG electrodes are 0.24 and 0.66 cm^2^, respectively.

Although some research groups continue to estimate the *A*_ele_ using the Randles–Sevcik equation, several studies have highlighted the significant uncertainties associated with this approach.^51–53^ This limitation occurs because the calculated area is highly dependent on the measurement time scale and becomes unreliable when applied to rough or porous electrodes.^53^ In this context, some theoretical electrochemists^51^ have suggested the estimation of electroactive area from the double-layer capacitance (*C*_dl_) as a more consistent strategy. Nevertheless, it is important to note that both approaches present intrinsic limitations, and under standard CV conditions, the electroactive area often approximates the geometric area.

To this end, *A*_e__l__e_ was estimated using the *C*_dl_ determined by cyclic voltammetry (CV) experiments, as described in the Experimental Section. Figure S11 presents the scan rate studies performed in this investigation. A linear relationship between the non-Faradaic current density (*j*) and the scan rate was observed, enabling calculation of *C*_dl_ from the slope of the resulting plot. Notably, the *C*_dl_ of the LIG electrode increased by a factor of around 26.7 following DMSO treatment, from 20.8 to 556 μF cm^–2^ for the bare and DMSO-treated LIG electrodes, respectively. High Cdl values have also been reported for LIG surfaces subjected to other chemical treatments. For instance, Reina et al.^54^ reported a specific capacitance of approximately 20 mF cm^–2^ for nitric acid-treated LIG, which is in line with the magnitude observed here. Such elevated values are typically attributed to the high porosity and increased surface area of the functionalized graphene structures.

By using the theoretical capacitance for graphene-based materials (*C*_s_ = 21 μF cm^–2^)^55^ and the geometric area of the electrode (*A*_geo_ = 0.19 cm^2^), the estimated electroactive areas were 0.18 and 5.0 cm^2^ for the bare and DMSO-treated LIG electrodes, respectively. The higher *A*_e__l__e_ observed for the DMSO-treated LIG electrode is consistent with the SEM images in Figure 3, which revealed a more dendritic surface texture and an increased number of surface capillaries following the treatment. These morphological changes likely contribute to the increase in the electroactive area, also observed by BET analysis.

Figure S12 shows the Nyquist plots obtained from EIS experiments for the LIG material before and after the DMSO treatment. While the bare LIG exhibits a semicircular profile indicative of charge-transfer-limited behavior, the Nyquist plot of the DMSO-treated LIG is almost vertical, reflecting a dramatic decrease in charge-transfer resistance (*R*_ct_). This qualitative observation is consistent with the enhanced electrochemical response observed in cyclic voltammetry measurements and confirms the effectiveness of the DMSO treatment.

Following this solvent treatment procedure, DMSO-treated LIG surfaces were evaluated as working electrodes for the detection of various compounds. Figure 7 presents the electrochemical profiles of several molecules, including SFL, chloramphenicol, sulfamethoxazole, ascorbic acid, tetracycline, amoxicillin, and ciprofloxacin, investigated by CV at a scan rate of 50 mV s^–1^, registered on bare and DMSO-treated LIG electrodes. As observed, the peak current values increased significantly after surface treatment, with enhancements ranging from 1.3- to 3.4-fold compared to the untreated electrodes.

**Figure 7:**
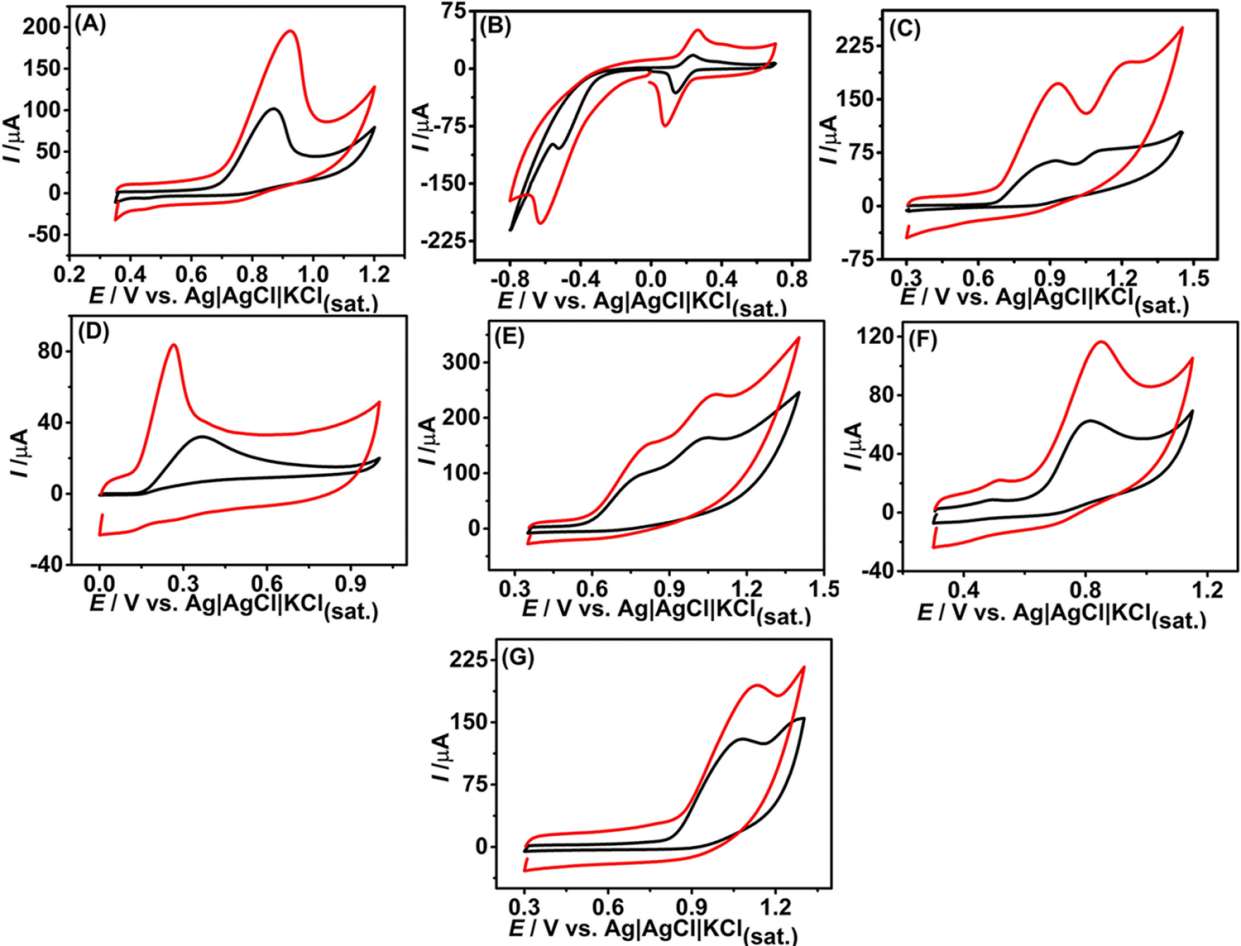
Cyclic voltammograms obtained for 1.0 mmol L^–1^ of different analytes in BR buffer solution at pH = 6.0: (A) SFL, (B) chloramphenicol, (C) sulfamethoxazole, (D) ascorbic acid, (E) tetracycline , (F) amoxicillin, and (G) ciprofloxacin, before (black line) and after (red line) electrode modification with DMSO. CV conditions: scan rate: 50 mV s^–1^ and step potential: 5 mV.

In the case of ascorbic acid, a negative shift in the oxidation peak potential was also observed following the DMSO treatment. This shift is likely due to hydrogen-bonding interactions between the lactone moiety of ascorbic acid and the oxygen-containing functional groups introduced onto the LIG surface during treatment, as confirmed by XPS analysis (Table S1). These interactions enhance the acidity of the hydroxyl protons within the ascorbic acid structure, thereby making the oxidation process more favorable.^56,57^

The enhanced electrochemical performance achieved with the treatment (as observed in CV and EIS data) can be linked to the increased number of oxygenated functional groups, as well as to structural defects, both of which contribute to improved charge transfer and faster HET kinetics.^56^

Similar trends were observed for other tested organic molecules, indicating that the presence of oxygen- and sulfur-containing groups on the LIG surface generally facilitates electrochemical reactions. In addition to promoting specific interactions, such as hydrogen bonding, these groups increase the hydrophilicity of the electrode surface, improving wetting and contact with aqueous analytes. Together with structural defects introduced during DMSO treatment, these factors enhance charge transfer and accelerate HET kinetics, contributing to overall improved sensor performance.

### Electrochemical Detection of SFL

To demonstrate the potential of DMSO-treated LIG electrodes for use in electroanalytical device development, we investigated the electrochemical behavior of SFL, a widely used antibiotic with applications across various fields.^58^ As previously observed in Figure 7A, the oxidation peak current increased from 74 ± 2 to 132 ± 6 μA upon DMSO treatment.

Initially, the redox behavior of SFL was assessed by cyclic voltammetry (CV) in Britton–Robinson (BR) buffer solutions across a pH range of 2.0–12.0, using a potential window from −0.65 to 1.45 V (Figure S13). In this potential range, a distinct and independent electrochemical process was identified, consistent with findings previously reported by Di-Oliveira and colleagues.^59^ As noticed, three electrochemical responses were observed in this study (*O*_x1_ at around +0.93 V, and two reduction processes (*R*_ed1_ at +0.35 V and *R*_ed2_ at +0.14 V). In addition, the BR buffer at pH 7.0 displayed higher current responses and a well-defined CV profile. Thus, this medium was selected as the supporting electrolyte for further studies.

As also observed in Figure S14, in the first cycle (sweeping in the anodic direction), an anodic peak (*O*_x1_) was observed at +0.93 V *vs.* Ag|AgCl|KCl_(sat.)_, corresponding to the oxidation of the phenylamine group into a radical species.^59^ This radical subsequently undergoes dimerization, forming an intermediate dimer, 4,4′-(hydrazine-1,2-diyl)­dibenzenesulfonamide. In the second cycle, the redox behavior of this dimer was observed, corresponding to the reversible transformation between 4,4′-(diazene-1,2-diyl)­dibenzenesulfonamide and 4,4′-(hydrazine-1,2-diyl)­dibenzenesulfonamide, as evidenced by the *O*_x3_/*R*_ed1_ redox couple at approximately +0.350 V, as reported in the literature.^59^ The reduction process (*R*_ed2_), observed at +0.14 V *vs.* Ag|AgCl|KCl­(sat.), appears to be dependent on the Red1 process and may be associated with the partial dissociation of the dimer, leading to the regeneration of the SFL species.^59^

The instability of the voltammetric signal for SFL at +0.93 V (*O*_x1_) (Figure S14), attributed to electrode surface fouling, limits the feasibility of its direct electrooxidation for analytical purposes. Conversely, the consistent and well-defined response of *R*_ed1_ (Figure S14) suggests that an indirect detection strategy may provide a more reliable alternative. As a result, further voltammetric investigations were carried out on this reduction process, *R*_ed1_, to better understand its potential for analytical applications.

Subsequently, studies were conducted using DPV scans to determine the optimal applied potential (Figure S15) and accumulation time (Figure S16) required to efficiently oxidize 5.0 μmol L^–1^ SFL and generate its corresponding oxidation product, which could then be reduced in the subsequent step. Thus, a better electrochemical response was achieved with the following conditions: applied potential = +1.0 V (*vs*. Ag|AgCl|KCl_(sat.)_) and accumulation time = 30 s.

The DPV parameters, including step potential, modulation amplitude, and modulation time, were evaluated using 5.0 μmol L^–1^ SFL in BR buffer (pH 7.0). The selection criteria for these parameters were based on precision, selectivity, maximum current response, well-defined voltammograms, and the highest analytical frequency. Thus, a step potential of 6 mV, a modulation amplitude of 80 mV, and a modulation time of 40 ms were selected, as shown in Figures S17–S19 and Table S2.

Under the optimized experimental conditions, the electrochemical sensor was evaluated for detecting SFL in water samples. The analytical curves were constructed using both bare and DMSO-treated LIG electrodes (Figure 8). A linear relationship between the cathodic peak current and the concentrations of the antibiotic (*R* ^2^ = 0.995) was obtained from 1.0 to 10.0 μmol L^–1^ using the unmodified electrode. On the other hand, utilizing the DMSO-treated LIG sensor, the calibration curve exhibited satisfactory linearity (*R* ^2^ = 0.997) over the concentration range from 0.3 to 9 μmol L^–1^. Table 1 shows some analytical parameters (linear range, limit of detection (LOD), and slope (sensitivity) values) using both electrodes. The LOD was estimated according to the IUPAC recommendation (3σ/s), where σ represents the standard deviation of the baseline noise, and *s* is the slope of the analytical curve (sensitivity).

**Figure 8:**
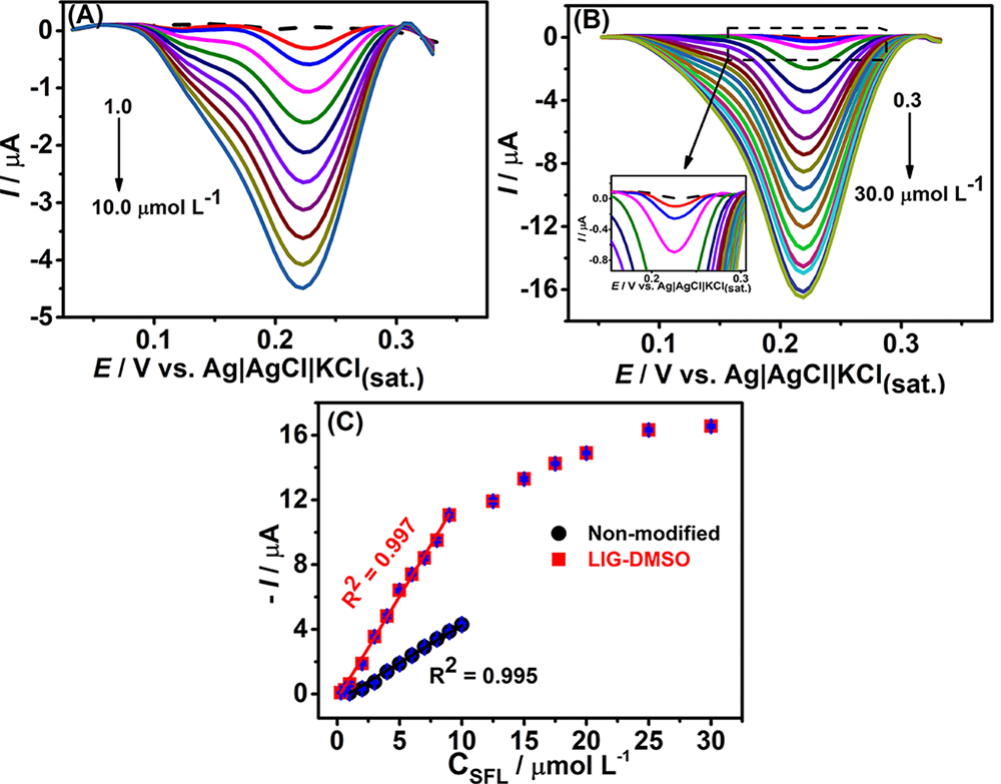
DPV responses (*n* = 3) for concentrations of SFL on (A) bare electrode (1.0–10.0 μmol L^–1^), (B) DMSO-treated LIG electrode (0.3–30.0 μmol L^–1^), and (C) respective calibration curves. Optimized experimental conditions are summarized in Table S2.

**Table 1:** Analytical Features of the DPV Method for SFL Detection on Both Bare and DMSO-Treated LIG Electrodes

analytical features	bare	modified
linear range (μmol L^–1^)	1.0–10.0	0.3–9.0
sensitivity (μA L μmol^–1^)	0.460 ± 0.004	1.270 ± 0.006
intercept(μA)	–0.39 ± 0.01	–0.31 ± 0.04
^a^LOD (μmol L^–1^)	0.3	0.09
LOQ (μmol L^–1^)	1.0	0.3
*R* ^2^	0.995	0.997

a The limit of detection (LOD) was calculated according to the IUPAC recommendation as LOD = 3σ/*s*, where σ is the standard deviation of the baseline noise, and *s* is the slope of the calibration curve.

As noticed, a considerable improvement in the sensitivity value was achieved (by 2.8 times) after the DMSO treatment; consequently, a lower LOD was also observed using DMSO-treated LIG electrodes. The intraelectrode precision (repeatability studies) after the treatment was conducted by successive DPV measurements of SFL at three concentration levels (2, 5, and 10 μmol L^–1^) (Figure S20). The relative standard deviation (RSD) values obtained were lower than 2.4%, indicating the good precision of this method. Additionally, the interelectrode precision (Figure S21) was also verified using three different (*n* = 3) DMSO-LIG electrodes in the presence of 2 μmol L^–1^ SFL. The cathodic peak current exhibited an RSD of 5.1%, indicating acceptable accuracy of the proposed method.

It is important to mention that bare LIG electrodes typically lack the sensitivity required for antibiotic detection in real samples. Here, a simple and low-cost DMSO treatment enhances the electrochemical activity of LIG, enabling sensitive and reliable detection, while preserving its fabrication simplicity.

The hysteresis of the proposed sensor was further investigated by testing sequential additions of SFL at different concentrations. The experiment was performed by first recording the signal at 1.0 μmol L^–1^, followed by 4.0 μmol L^–1^, and subsequently in reverse order (4.0 and 1.0 μmol L^–1^), Figure S22. In both cases, the recorded currents were consistent within experimental error (RSD < 10%), indicating that the sensor response is stable, reproducible, and does not exhibit hysteresis effects.

Additionally, the proposed sensor (fabricated by CO_2_ laser irradiation on polyimide sheets and subsequently modified with DMSO) was compared to other electrochemical sensors based on key analytical parameters (LOD, linear range, and sensitivity) as well as production cost. The DMSO-treated LIG electrode exhibited electrochemical performance comparable or superior to the most previously reported sensors for SFL detection (Table S3), including those based on conventional electrodes such as glassy carbon.^60^

While Wei et al.^61^ report a higher sensitivity, it employs a molecularly imprinted polymer (MIP) layer, which typically involves multistep procedures, higher cost, and reduced scalability. Lisboa and collaborators^62^ present a lower LOD using 3D-printed electrodes. However, as observed in both Rocha et al.^63^ and Lisboa et al.,^62^ the electrochemical performance varies significantly among similar devices, revealing a lack of reproducibility often associated with 3D printing techniques. In contrast, the fabrication of LIG electrodes is scalable, rapid, direct, and residue-free, requiring no additional chemical reagents or postprocessing. The DMSO modification is also extremely simple, using only a small volume (3 μL) of solvent. Therefore, beyond its competitive analytical performance, the proposed sensor stands out due to its practical simplicity, low cost, and fabrication reproducibility, making it a promising platform for real-world electrochemical sensing applications, particularly for SFL detection in water samples.

In addition, the analytical performance of DMSO-treated LIG sensors was compared to that of a conventional glassy carbon electrode (GCE) under identical experimental conditions. As shown in Table S4 and Figure S23, the LIG sensor exhibited superior performance, with the slope of the calibration curve (sensitivity) increasing from 0.16 to 6.7 μA cm^–2^ μmol^–1^. All values were normalized to the geometric area of the electrodes to ensure a fair comparison. These results highlight that the DMSO-treated LIG electrodes provide enhanced sensitivity and competitive analytical performance relative to conventional GCE, demonstrating the effectiveness of the surface modification.

### Interfering Species

Antibiotics are considered emerging environmental pollutants, and certain metal ions are also commonly found in water samples.^64–66^ Therefore, the selectivity of the proposed electrochemical method was evaluated in the presence of an equimolar mixture (5.0 μmol L^–1^) of SFL and potential interfering substances, including Cu^2^^+^, CIP, TC, K^+^, AMX, CAP, Cd^2^^+^, Mg^2+^, Na^+^, Ca^2+^, NO_3_^–^, sulfamethoxazole (SFZ), and PAR (Figure S24). The recovery rates obtained for SFL after the addition of each interferent ranged from 96% to 104%, as shown in Figure S24, indicating no significant interference and confirming the high selectivity of the proposed method. Although sulfamethoxazole exhibits a reduction peak close to that of SFL, the peaks are sufficiently separated (at around ∼100 mV), allowing the accurate determination of SFL in its presence.

### Determination of SFL in Environmental and Biological Samples

As a proof-of-concept, the accuracy of the proposed method for SFL quantification in environmental (river, drinking, and tap water) and biological (synthetic urine) samples was evaluated under optimized DPV conditions using a DMSO-treated LIG electrode. Tap and drinking water samples were spiked with known amounts of SFL at two concentration levels (1 and 4 μmol L^–1^), and the recovery tests were performed using the standard addition method, as noticed in Figures S25 and S26. In addition, synthetic urine and river samples were spiked with 1 and 2 μmol L^–1^, as observed in Figures S27 and S28. These results are summarized in Table 2.

**Table 2:** Concentrations and Recovery Values Obtained for the Analysis of Water Samples (*n* = 3)

sample	spiked (μmol L^–1^)	found ± SD (μmol L^–1^)	recovery ± SD (%)
tap water	0.0	<LOD	
	1.0	1.00 ± 0.04	100 ± 4
	4.0	3.9 ± 0.1	98 ± 3
drinking water	0.0	<LOD	
	1.0	1.04 ± 0.03	104 ± 3
	4.0	4.1 ± 0.1	103 ± 3
river water	0.0	<LOD	
	1.0	1.0 ± 0.1	103 ± 5
	2.0	1.9 ± 0.1	97 ± 1
synthetic urine	0.0	<LOD	
	1.0	0.9 ± 0.1	90 ± 4
	2.0	1.9 ± 0.1	97 ± 5

According to the IUPAC guidelines, in the absence of certified reference materials, accuracy can be evaluated through spiking and recovery studies. In this approach, a test sample is analyzed both before and after the addition of a known amount of analyte, and the difference is used to calculate the recovery. Therefore, recovery experiments are internationally recognized as a suitable means to assess the method accuracy in complex matrices.

As observed, before spiking, no analytical signals (<LOD) were detected close to the SFL peak position in any of the samples. Following the analysis of the spiked samples, the obtained recovery values (90**–**104%) demonstrated the high accuracy of the proposed method, which was free from interference from both sample matrices.

In addition, the results obtained with the proposed method were statistically compared with those from a validated spectrophotometric method at one concentration level (Table S5). *t*- and *F*-tests (*n* = 3) yielded *t* values of 0.37–1.78 (*t*_crit_ = 2.776) and *F* values of 1.56–11.11 (*F*_crit_ = 19), indicating no significant differences between the means or variances of the two methods with 95% confidence levels.

Therefore, these results confirm that the proposed DPV method provides accurate and statistically comparable results to those of the reference spectrophotometric procedure described in the literature, supporting its applicability for SFL determination in both environmental and biological matrices.

## Conclusions

We show for the first time that the application of only 3.0 μL of DMSO to the surface of the LIG electrodes significantly enhances their electrochemical activity. This improvement is attributed to changes in the surface composition of the material, specifically the introduction of C=O and π–π* functionalities following solvent treatment, as confirmed by XPS analysis. In addition, the application of DMSO significantly increases the surface hydrophilicity, as evidenced by contact angle measurements, and contributes to an enlarged electroactive area.

We also demonstrated the potential of the modified electrode for the detection of various analytes, including ciprofloxacin, ascorbic acid, SFL, and tetracycline. As a proof-of-concept, the sensor was successfully applied to determine SFL in synthetic urine and water samples using DPV scans. After electrochemical treatment, the sensitivity (i.e., slope of the calibration curve) increased from 0.460 ± 0.004 to 1.270 ± 0.006 μA μmol^–1^ L.

We believe that the modified LIG electrodes not only hold great promise for the advancement of electrochemical sensing platforms but also open exciting possibilities for integration into a wide range of optoelectronic applications, including supercapacitors, SERS substrates, and next-generation functional devices.

Despite these promising results, several limitations should be noted. The use of DMSO, although minimal (3 μL per electrode), involves handling a toxic solvent. The performance of LIG electrodes also depends on the quality of the laser source and the type and thickness of the polyimide precursor, which may affect reproducibility. In addition, the electrodes are intended as disposable devices, such that mechanical detachment of the LIG structure can occur after disconnection from the connectors, and the DMSO treatment induces irreversible surface modifications, preventing regeneration. At higher concentrations, SFL also shows strong adsorption onto the LIG surface, which further compromises the reusability. Finally, while DMSO clearly enhances electrochemical activity, it may also contribute to surface functionalization, and exploring alternative solvents or modification strategies could open up new possibilities for tuning electrode properties. Future work should address these factors to expand the applicability and safety of LIG-based electrochemical platforms.

## Supplementary Material



## Notes

Supporting Information provides additional experimental details, including materials and methods, electrode characterization (electrochemical impedance spectroscopy, SEM, contact angle, Raman, and XPS analyses of bare and DMSO-treated LIG electrodes), optimization of the proposed DPV method, inter- and intraelectrode precision studies, comparison with literature data, and accuracy evaluation through recovery experiments using the spectrophotometric reference method (PDF)
