# Supplementary material for: Solvent-Assisted Modification of Laser-Induced Graphene for Surface-Enhanced Electrochemical Response
Source: Anal Chem. 2026 Jan 20;98(4):3103–15. doi: 10.1021/acs.analchem.5c06456 (PMC12874222; doi:10.1021/acs.analchem.5c06456)
Supplement: Supplementary file 1 [file ac5c06456_si_001.pdf]

Supporting information
Inoque *et al.*

Solvent-assisted modification of laser-induced graphene for surface-enhanced electrochemical response

Nélio I. G. Inoque[a], Raquel G. Rocha[a], Gilvana P. Siqueira[a], Ana Clara Maia Oliveira[a], Michele V. C. O. Da Silva[a], Robert D. Crapnell[b], Craig E. Banks[b], Edson Nossol[a], Eduardo Mathias Richter[a] and Rodrigo A. A. Muñoz[a*]

[a]*Institute of Chemistry, Federal University of Uberlândia, 38400-902, Uberlândia, Minas Gerais, Brazil*

[b]*Faculty of Science and Engineering, Manchester Metropolitan University, Dalton Building, Chester Street, M1 5GD, Great Britain*

***Corresponding author:** munoz@ufu.br

Table of content

Experimental Section……………………………………………………………S2

Reagent and Samples………………………………………………………………...S2

Morphological and spectroscopic characterizations…………………………………...S4

Electrochemical measurements……………………………………………………...S5

Spectrophotometric measurements…………………………………………………..S7

Results and discussion……………………………………………………………...S8

Optimization of proposed treatment……………………………………………..……...S8

Morphological and spectroscopic characterization……………………………………S9

Electrochemical characterization…………………………………………………..S14

Sulfanilamide detection…………………………………………………………….S17

References…………………………………………………………………………S34

Experimental Section

Reagents and samples

All reagents exhibited a high degree of analytical purity and were used without further purification. Acetic acid (99.7% w/v), dimethyl sulfoxide (99.8% w/w), acetonitrile (99.8% w/w), sodium chloride (99.8% w/w), citric acid (≥98 % w/w), ascorbic acid (99 % w/w) and sodium sulfate (99 % w/w), sulphuric acid (98% w/v), 8-hydroxyquinoline (98.5%) were purchased from Vetec® (Rio de Janeiro, Brazil); ethanol (95% v/v) was obtained from Dinâmica® (Indaiatuba, Brazil); methanol (100% w/w) from Honey well (Muskegon, USA); calcium chloride dihydrate (99.7% w/w), sodium hydroxide (98% w/w) and phosphoric acid (85% w/v), sodium nitrite (97% w/w) from Synth® (São Paulo, Brazil); boric acid (99.9% w/w) from Acros Organics® (New Jersey, USA); magnesium sulfate (97 % w/w), sodium nitrate (99.8% w/w) obtained from Merck (Darmstadt, Germany); magnesium sulfate heptahydrate (99.8% w/w) and ammonium chloride (99 % w/w) from Cinética Química (São Paulo, Brazil); potassium chloride (99.5% w/w) from Carlo Erba® (Cornaredo, Italy); dimethylformamide (99.8 % w/w) purchased from Êxodo Científica® (Sumare, Brazil); chloramphenicol (≥ 98 % w/w), amoxicillin (98.6 % w/w), tetracycline (≥98 % w/w), sulfanilamide (≥ 98 % w/w), uric acid (99 % w/w) and urea (99 % w/w) from Sigma-Aldrich® (St. Louis, USA); sulfamethoxazole (99% w/w) from Sinergia Científica® (Campinas, Brazil) and sodium hydrogen carbonate (99.5 % w/w); monopotassium phosphate (98 % w/w) and calcium chloride (99 % w/w) from Labsynth (Diadema, Brazil). Standard solutions of copper (II), potassium (I), and cadmium (II) nitrates, each at a concentration of 1000 $mg \cdot L^{-1}$, were obtained from SpecSol® (São Paulo, Brazil). All aqueous solutions were prepared using deionized water (resistivity ≥ 18 $M\Omega \cdot cm$) obtained from a Milli-Q water purification system (Millipore, Bedford, MA, USA).

Britton-Robinson (BR) buffer (pH = 2.0 - 10.0), composed of acetic, boric, and phosphoric acids each at a concentration of 0.04 mol·L^{-1}, was prepared by dissolving the appropriate amounts of these acids in deionized water. The pH was adjusted by the addition of a 1.0 mol L^{-1} NaOH solution. Stock solutions of sulfanilamide (SFL) were freshly prepared daily in BR buffer at pH 7.0.

Tap and drinking water were collected from our laboratory (Uberlândia, Brazil) and river water sample were collected from the Uberabinha River in Uberlândia, Minas Gerais, Brazil. Before analysis, appropriate concentrations reagents required to prepare the supporting electrolyte was added in the samples. Finally, the spiked tap and drinking water samples were placed into the electrochemical cell for the SFL electroanalysis, without the need for an additional dilution step, whereas fortified river water samples were diluted (4-fold) in BR buffer (pH 7.0) prior to analysis.

Synthetic urine was prepared following the protocol described by Brooks and coauthor [1], by dissolving 0.40 g of citric acid, 0.37 g of ascorbic acid, 2.10 g of sodium hydrogen carbonate, 9.99 g of urea, 0.07 g of uric acid, 0.04 g of calcium chloride, 5.20 g of sodium chloride, 1.41 g of sodium sulfate, 0.49 g of magnesium sulfate, 1.30 g of ammonium chloride, and 0.95 g of monopotassium phosphate in 1 L of deionized water. The synthetic urine sample fortified with sulfanilamide was subsequently diluted (10-fold) in BR buffer solution before electroanalysis. The samples were fortified with known amounts of SFL at two different concentration levels (1.0 and 2.0 or 4.0 µmol L^{-1}) for recovery studies by standard addition method.

Morphological and spectroscopic characterizations

Morphological characterization of the laser-induced graphene (LIG) electrodes was carried out using a scanning electron microscope (SEM, Vega3, Tescan, Czech Republic) operated at 20 kV with a working distance (WD) set to 15 mm. Elemental composition was assessed via energy-dispersive X-ray spectroscopy (EDS) using an INCA X-Act detector (Oxford Instruments, UK). To prevent structural degradation under the electron beam, all samples were sputter-coated with a ~10 nm gold layer prior to SEM and EDS analysis. Specific surface area measurements were performed by nitrogen physisorption at 77 K using a Micromeritics ASAP 2000 instrument (Norcross, GA, USA). Samples were degassed at 80 °C under vacuum (<10 µmHg) before analysis. Surface area values were calculated using the Brunauer–Emmett–Teller (BET) model. Topographic and roughness analysis was conducted using an atomic force microscope (AFM, SPM-9600, Shimadzu, Kyoto, Japan) equipped with a standard scanner, capable of imaging areas of 10 × 10 µm with a vertical range of 5 µm. The AFM tips exhibited a curvature radius below 10 nm and operated within a frequency range of 204–497 kHz. The Z-axis scale was set to 1 µm. Surface roughness parameters were extracted using Gwydion software. The crystal structure, surface morphology, and chemical composition of the prepared materials were thoroughly analysed. These included powder X-ray diffraction (XRD 6000 diffractometer from Shimadzu (Japan), Cu Kα radiation, λ = 1.5406 Å).

The Raman spectra of the LIG electrodes were performed using a Confocal Raman Microscope LabRAM HR Evolution (Horiba, Kyoto, Japan), equipped with a green laser (532 nm) emitting 2 mW of power. Surface contact angle images of both unmodified and solvent-treated LIG electrodes were captured using a smartphone mounted on a universal holder. The images were taken 10 s after a drop of deionized water was placed on the surface of the working electrode. The contact angle, defined as the angle between the

tangent at the liquid–air interface of the droplet and the electrode surface, was measured using GeoGebra® software (https://www.geogebra.org/classic).

X-ray Photoelectron Spectroscopy (XPS) measurements were performed using an AXIS Supra system (Kratos, UK) equipped with a monochromatic Al Kα X-ray source (1486.6 eV) operating at 225 W and a hemispherical sector analyzer. Data was acquired in fixed transmission mode, with pass energies of 160 eV for survey spectra and 20 eV for high-resolution scans. The analysis was conducted in slot mode, yielding a sampling area of approximately 700 × 300 µm. Under a pass energy of 20 eV, the full width at half maximum (FWHM) of the Ag $3d_{5/2}$ peak was measured at 0.613 eV. The binding energy scale was calibrated using the sp^2-hybridized graphitic C 1s peak, set at 284.5 eV. Despite known limitations associated with this calibration method,[2] it was employed due to the unavailability of more suitable reference standards and the limited dependence of the analysis on absolute peak positions.

Electrochemical measurements

All voltammetric measurements were carried out employing a µ-AUTOLAB Type III potentiostat/galvanostat (Metrohm Autolab BV, Utrecht, the Netherlands), coupled to a computer and controlled by NOVA 2.1.7 software. A 3D-printed electrochemical cell (internal volume of 10 mL), as prototyped by Cardoso and coauthors,[3] was used for cyclic voltammetry (CV) and differential pulse voltammetry (DPV) measurements (Figure 1G). In our setup, the geometric area of the working electrode was defined by a rubber O-ring positioned between the electrode surface and the cell body. This O-ring ensures that only the circular area (0.19 cm^2) remains exposed to the supporting electrolyte. In addition, the O-ring provides an effective mechanical seal, preventing any leakage or lateral contact of the solution with non-active electrode regions.

The reference and auxiliary electrodes were Ag|AgCl|$KCl_{(sat.)}$ and a platinum wire, respectively. The LIG electrode, before and after solvent treatment, was employed as the working electrode. A glassy carbon electrode (GCE) with a diameter of 3 mm (geometric area of 0.07 cm^2), purchased from BAS Inc. (West Lafayette, USA), was also employed as a working electrode for comparative evaluation. All electrochemical data were performed in the presence of dissolved oxygen and at room temperature (~25 °C).

The CV data were used to estimate the double-layer capacitance (*C*dl) within a potential window of 0.0 to +0.3 V (*vs*. Ag|AgCl|$KCl_{(sat.)}$ at different scan rates in a 0.10 mol·L^{-1} KCl solution, chosen to avoid faradaic processes and ensure that the measured current corresponds exclusively to the double-layer charging. The use of KCl as an inert supporting electrolyte was intended to provide ionic conductivity without interfering chemically with the electrode material, preserving its surface properties. The procedure follows the methodology described by Voiry *et al.*.[4] The difference between the anodic and cathodic currents was measured at +0.15 V (*vs*. Ag|AgCl|KCl saturated), normalized by the geometric area (0.19 cm^2), and plotted against the scan rate (V s^{-1}) to determine *C*dl from the slope. The electroactive area (A_{ele}) was then estimated using a specific capacitance of 21 μF·cm^{-2} (theoretical surface capacitance, *Cs*) and the geometric area (A_{geo} = 0.19 cm^2), according to Equation (1):

$$A_{ele} = \frac{C_{dl}\ A_{geo}}{Cs} \quad (1)$$

Finally, as a proof of concept, the LIG-DMSO electrode was applied for the determination of SFL in a water sample under optimized DPV conditions (modulation amplitude = 80.0 mV; step potential = 6.0 mV; modulation time = 40.0 ms).

Electrochemical impedance spectroscopy (EIS) measurements were conducted at the open circuit potential (+0.22 V *vs*. Ag|AgCl|$KCl_{(sat.)}$) in the presence of a 1.0 mmol L^{-1} $[Fe(CN)_6]^{3-/4-}$ redox couple in a 0.10 mol L^{-1} KCl solution. An alternating potential with

an amplitude of 10 mV in a frequency range from 50,000 Hz to 0.1 Hz was applied during EIS experiment.

Spectrophotometric measurements

The spectrophotometric measurements were carried out using a Shimadzu UV–*vis* spectrophotometer (UV-2600 model) at a wavelength of 482 nm, employing 3.0 mL quartz cuvettes. The instrumental conditions and procedures were adapted from Nagaraja and colleagues.[5] Briefly, aliquots of standard SFL solutions were transferred into 25-mL flasks, followed by the addition of 1.0 mL of sulfuric acid (10 mol L^{-1}). After cooling in an ice bath, 1.5 mL of sodium nitrite (1.0% m/v) was added with gentle swirling to promote the diazotization reaction involving the primary amine group of SFL and $NaNO_2$ in an acidic medium. The solutions were allowed to stand for 5 min, after which 2.5 mL of sulfamic acid (2.0% w/v) was added, swirled, and left to stand for another 5 min. To promote the coupling of the diazotized form with 8-hydroxyquinoline (8-HQ) in an alkaline medium to produce a colored product, 2.0 mL of 8-HQ (0.5% w/v) and 2.0 mL of sodium hydroxide (5 mol L^{-1}) were then added. The volume was brought to 25 mL with Milli-Q water, and the solutions were mixed thoroughly. The absorbance of the colored reaction product was measured at 482 nm against a reagent blank after 5 min, and a calibration curve was constructed. This curve was used to quantify SFL in fortified and diluted samples, including tap and drinking water (2-fold diluted) and river water (4-fold diluted). In addition, the standard-addition method was applied to the determination of SFL in a 10-fold diluted urine sample in order to minimize matrix interferences.

Supporting information
Inoque *et al.*

Results and discussion

Optimization of proposed treatment

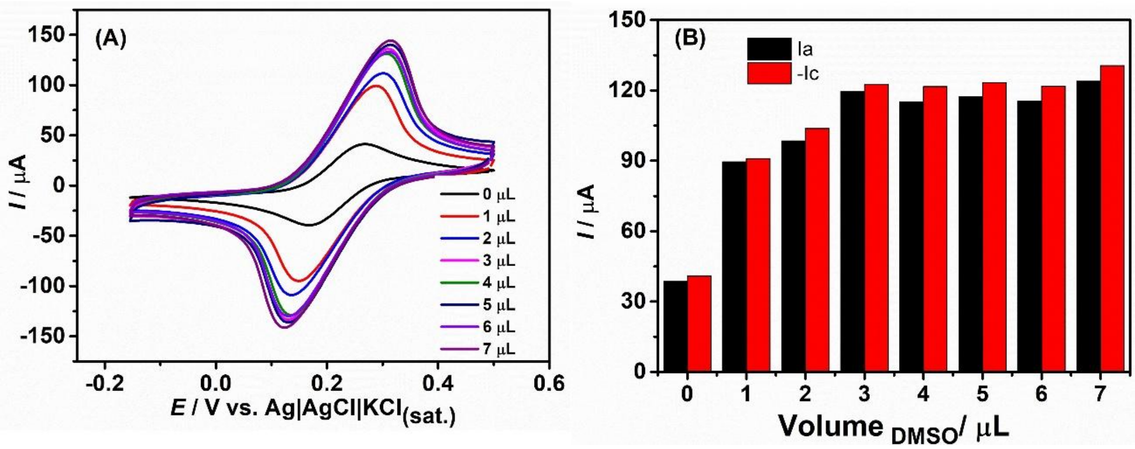

Figure S1. (A) Cyclic voltammograms recorded in the presence of 1 mmol L^{-1} $[Fe(CN)_6]^{3-/4-}$, in 0.10 mol L^{-1} KCl solution, using bare electrode (without DMSO, black line) and DMSO-modified LIG electrodes (different volumes ranging from 1 to 7 μL); (B) Anodic (Ia, black bar) and cathodic (-Ic, red bar) currents extracted from data shown in Figure **S1A**. **CV conditions:** step potential = 5 mV; scan rate: 50 mV s^{-1}.

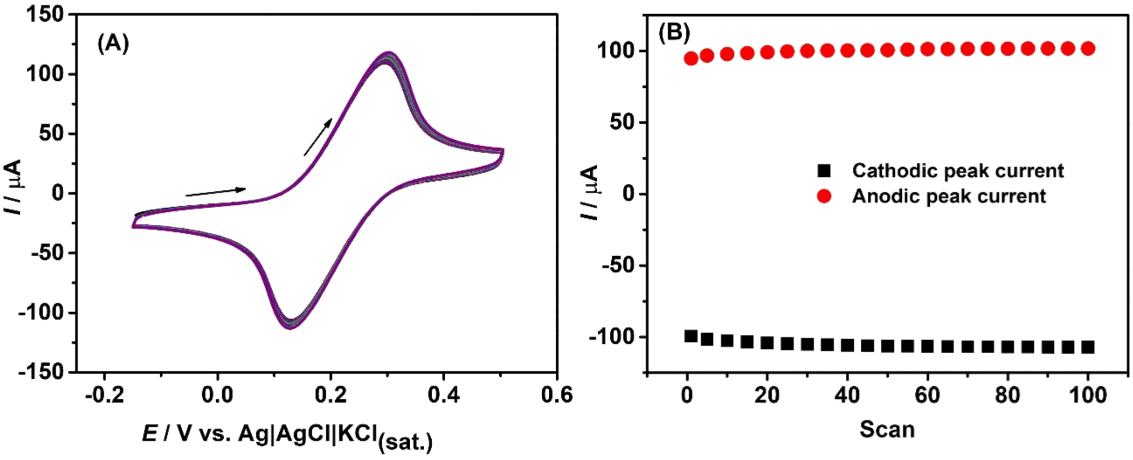

Figure S2. (A) CV voltammograms obtained from successive scans (n=100) in presence of 1.0 mmol L^{-1} $[Fe(CN)_6]^{3-/4-}$ in 0.1 mol L^{-1} KCl solution, using LIG-DMSO electrode; (B) Peak current variation of cathodic (squares) and anodic (circles) responses. **CV conditions:** step potential = 5 mV; scan rate: 50 mV s^{-1}.

Morphological and Spectroscopic characterizations

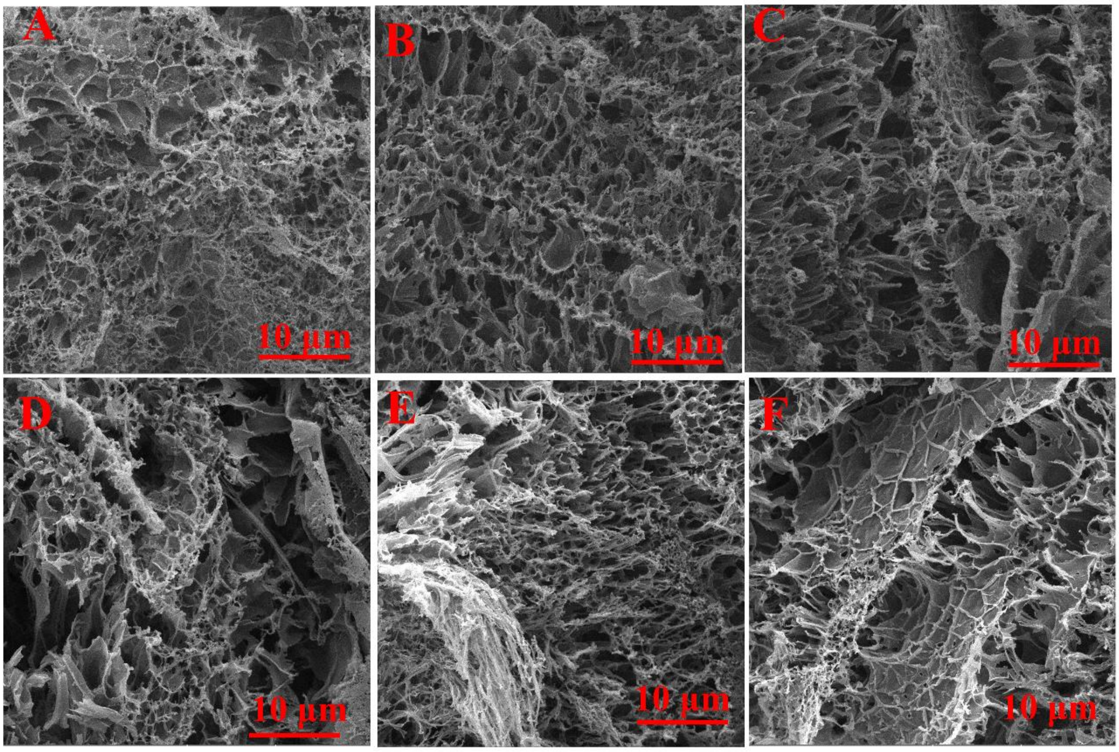

Figure S3. SEM images of the (A) unmodified LIG electrode and LIG electrodes modified with (B) DMSO; (C) EtOH; (D) MeOH; (E) ACN and (F) DMF.

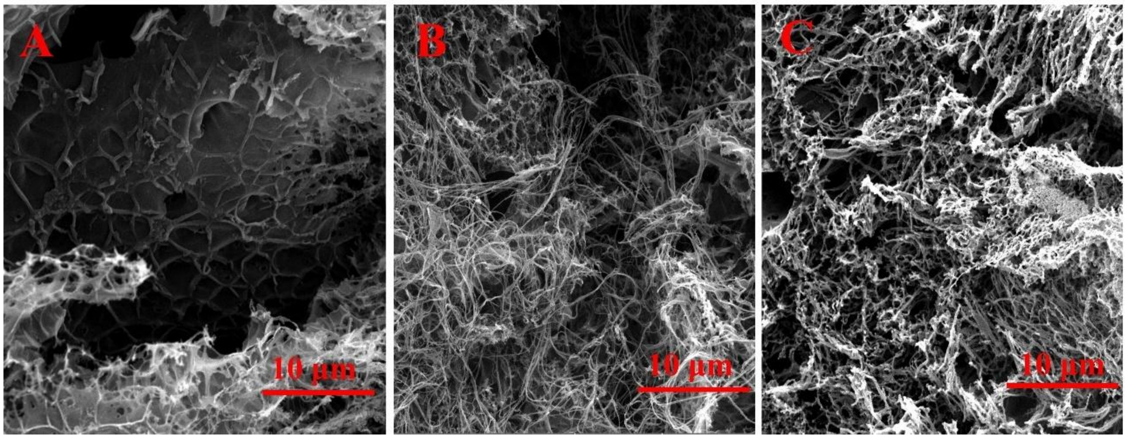

Figure S4. SEM images of LIG electrodes: (A) untreated surface, (B) LIG-DMSO, and (C) LIG-EtOH electrode surfaces.

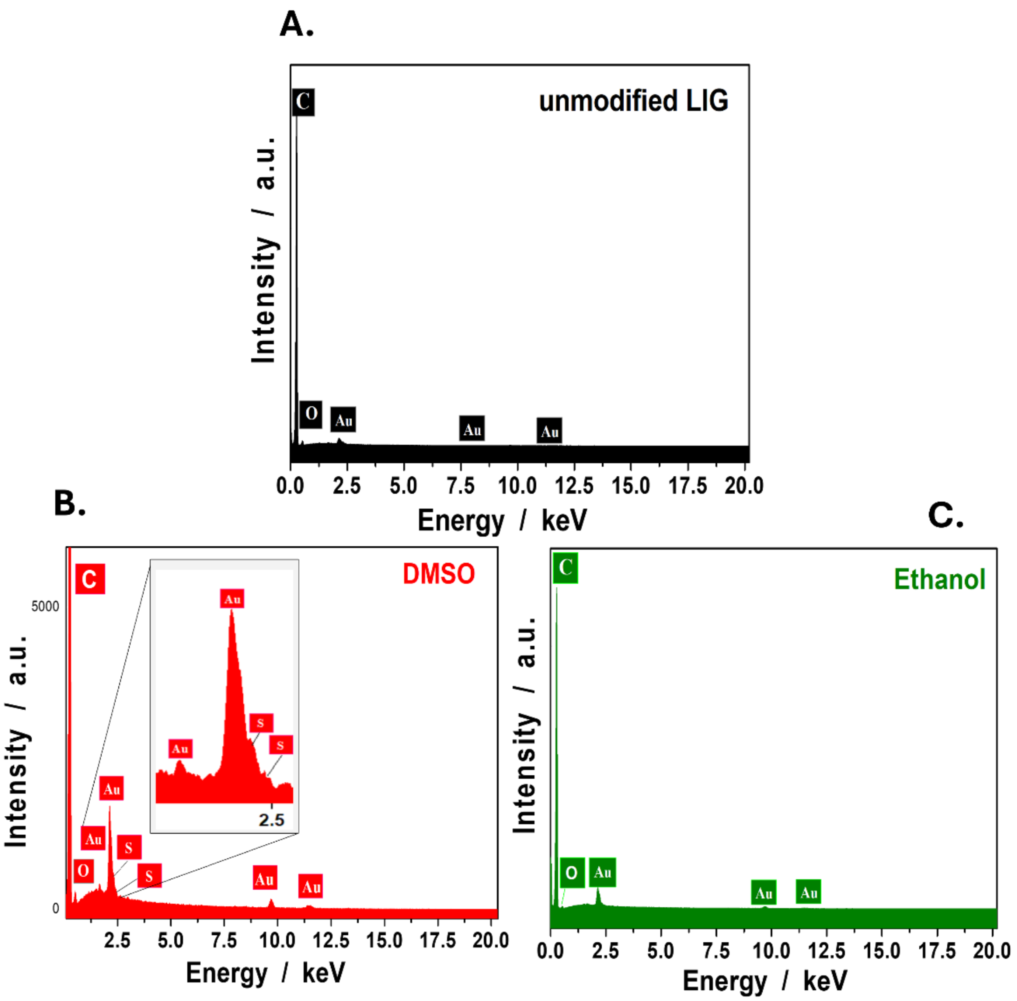

Figure S5. Energy-dispersive X-ray spectroscopy (EDS) spectra of the electrode surfaces: (A) unmodified LIG electrode, (B) LIG electrode after DMSO treatment, and (C) LIG electrode after ethanol treatment.

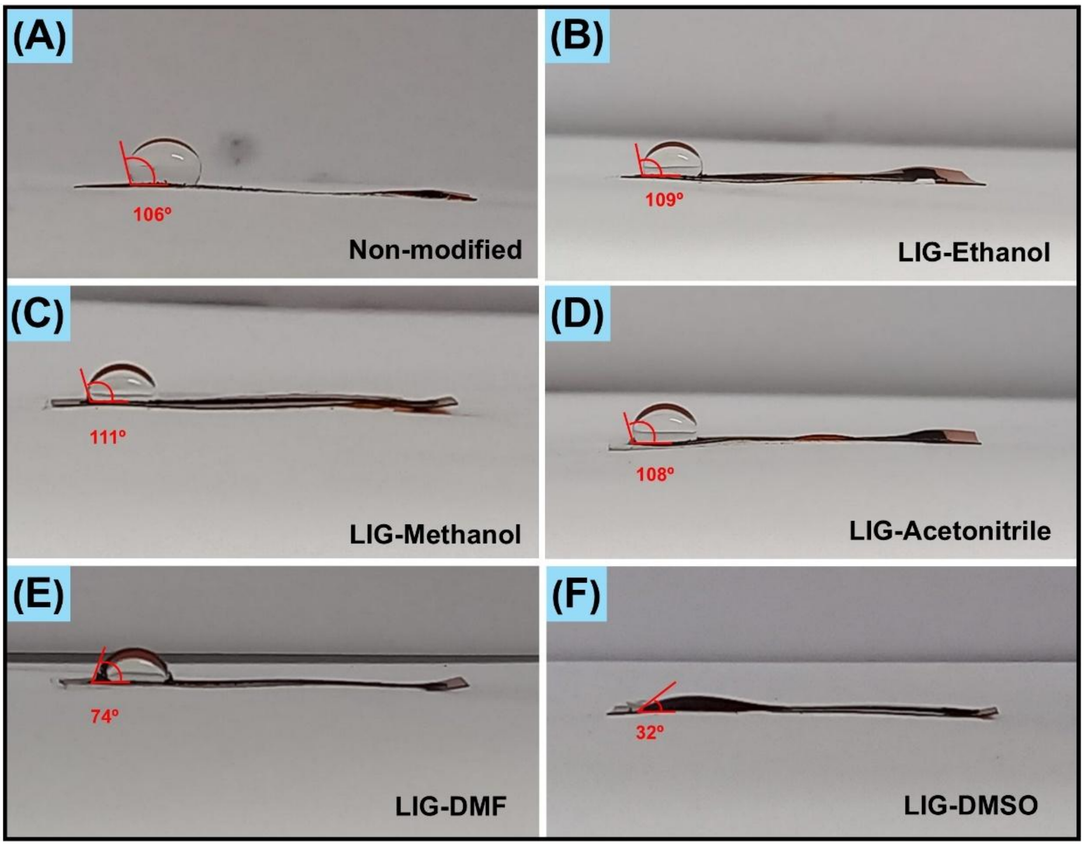

Figure S6. Contact angle images of (A) non-modified LIG electrodes and LIG electrodes modified with (B) ethanol, (C) methanol, (D) acetonitrile, (E) DMF, and (F) DMSO.

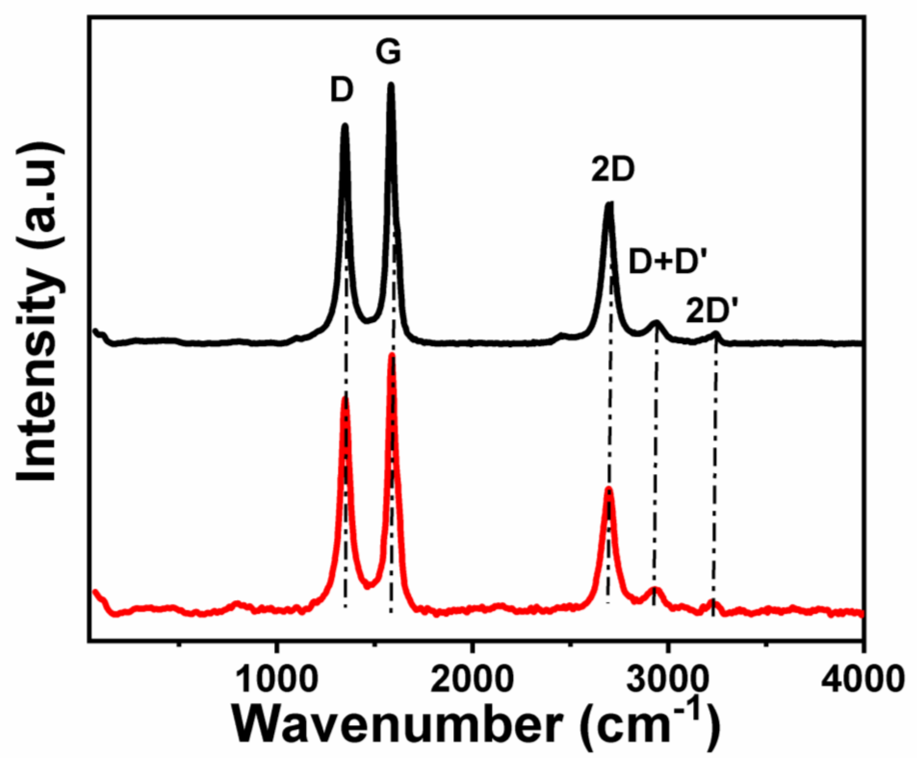

Figure S7. Raman spectra of the unmodified LIG electrode (black line) and the LIG electrode surface modified with DMSO (red line).

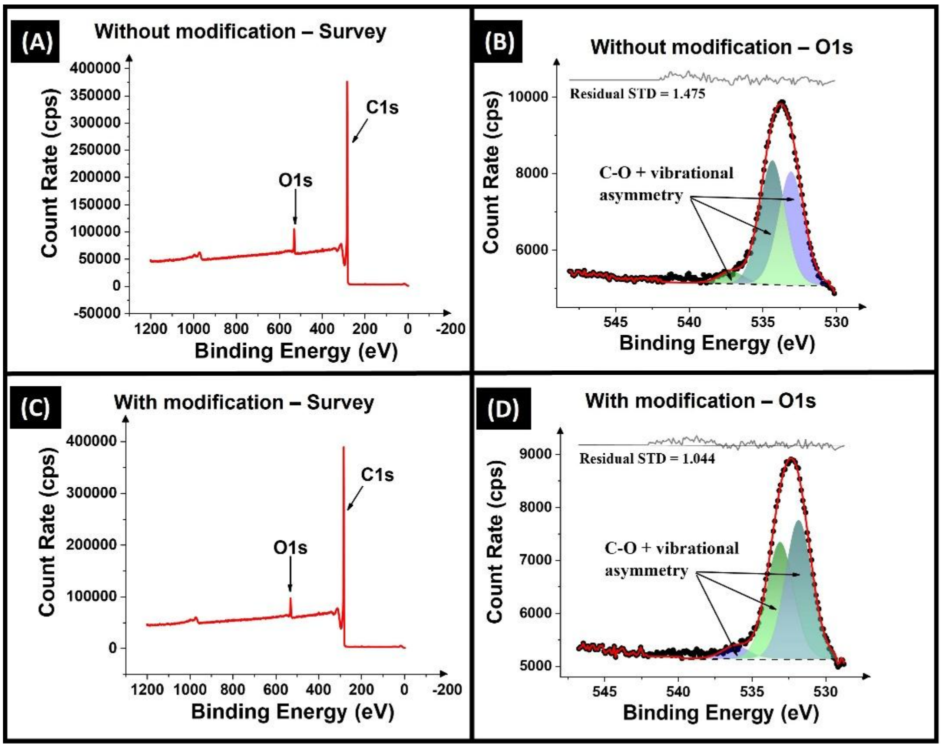

Figure S8. (A, C) XPS survey spectra and (B, D) high resolution deconvoluted O1s spectra obtained for bare and DMSO-treated LIG electrodes, respectively.

Table S1. XPS deconvoluted components of C1s spectra

	**Position (eV)**	**Composition (%)**	
		**Bare LIG**	**DMSO-LIG**
**Graphitic**	284.5	63.14	66.02
**C-C, C-H**	286.14	11.43	6.37
**O-C=O**	290.80	6.46	6.40
**C=O**	288.86	5.72	6.96
**C-O**	287.00	10.63	11.58
**π-π**	292.44	2.62	2.67

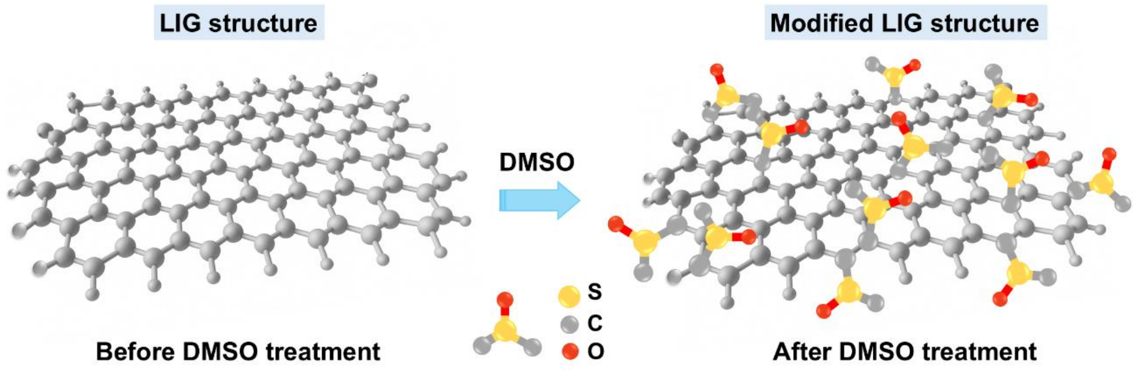

Figure S9. Schematic illustration of morphological surface modification on the LIG electrode before (bare) and after DMSO treatment (DMSO-treated electrode).

Electrochemical Characterization

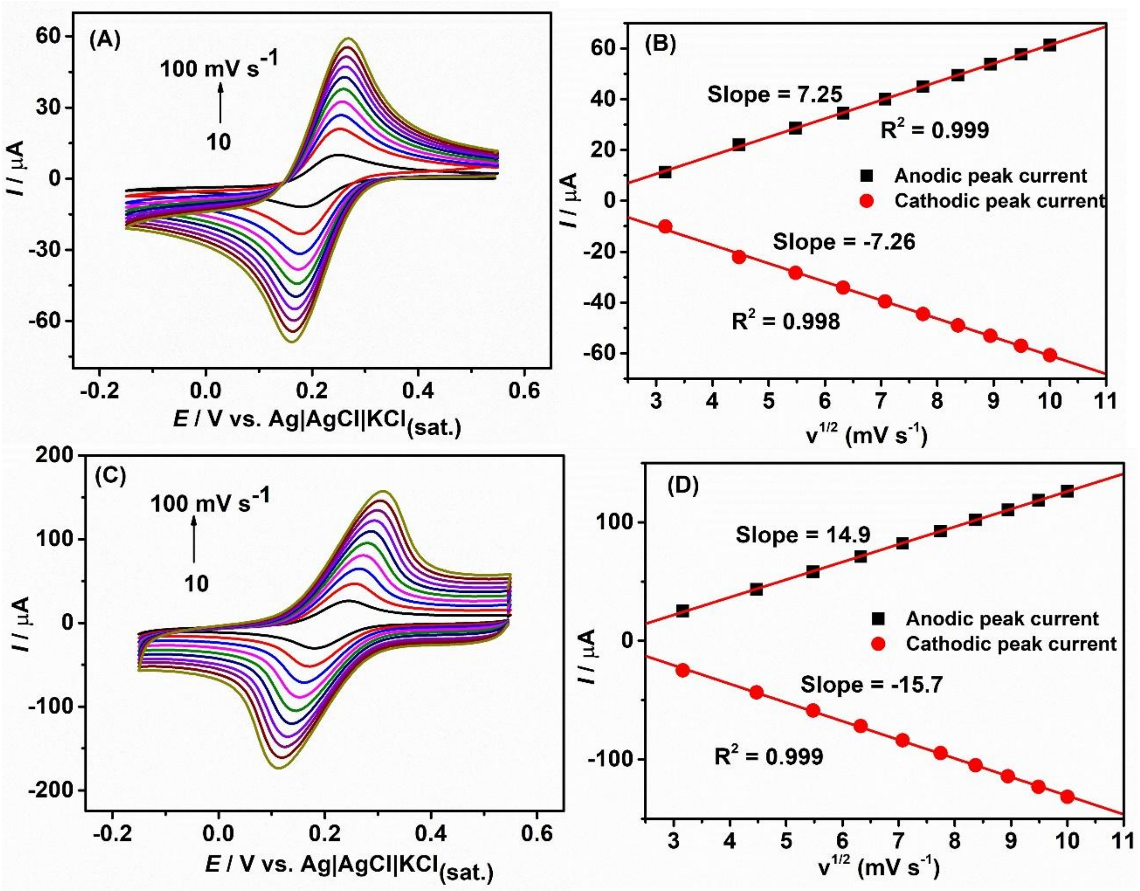

Figure S10. Cyclic voltammograms obtained for $[Fe(CN)_6]^{3-/4-}$ (1.0 mmol L^{-1}) in 0.1 mol L^{-1} KCl solution at different scan rates (10 - 100 mV/s) on (A) non-modified and (B) modified electrode; (C) and (D) Plots of anodic and cathodic peak currents as a function of the square root of the scan rate.

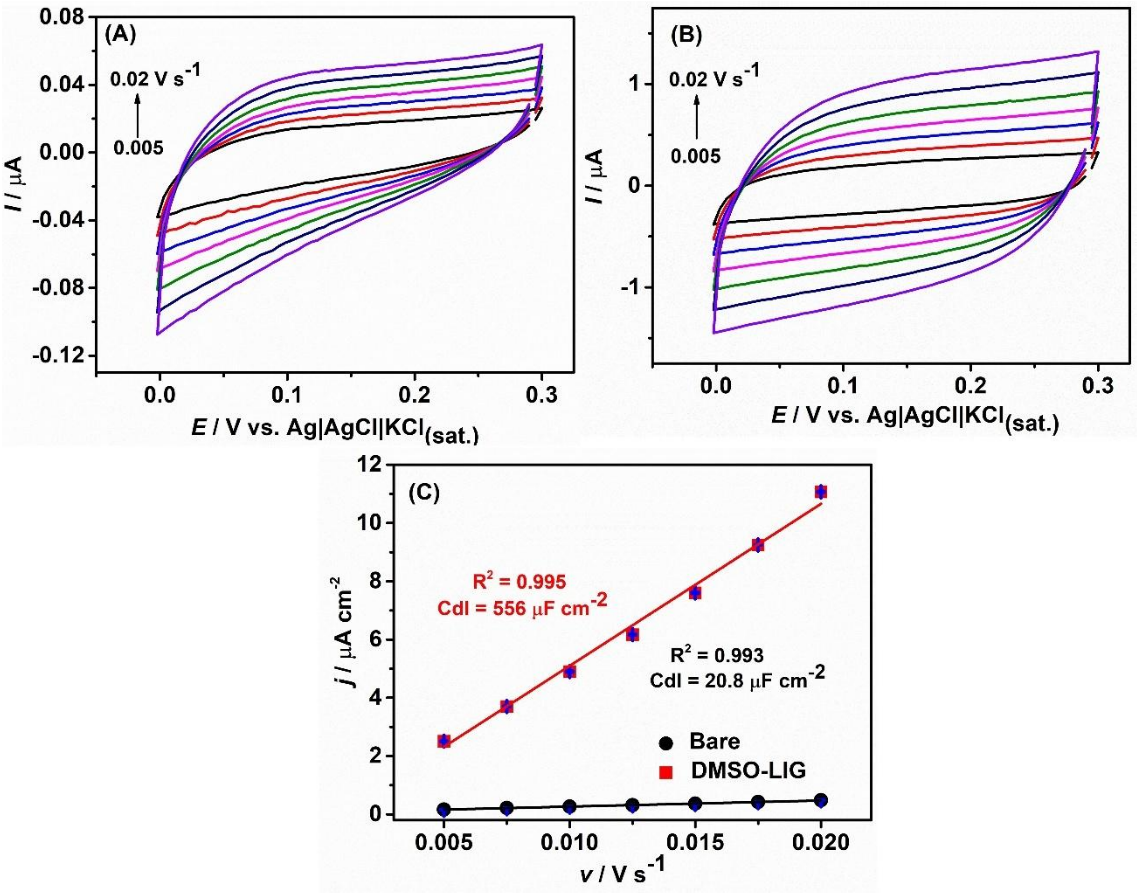

Figure S11. CV recorded at 10 to 30 mV s^{-1} at the (A) unmodified and (B) DMSO-LIG electrodes. CVs were measured in 0.1 mol L^{-1} KCl solution from 0.0 to +0.3 V *vs.* Ag|AgCl|$KCl_{(sat.)}$. (C) Capacitance data: plots of *j* (current density) measured at +0.15 V (*vs.* Ag|AgCl|$KCl_{(sat.)}$) *versus* CV scan rate on unmodified and modified electrodes to determine the Cdl value.

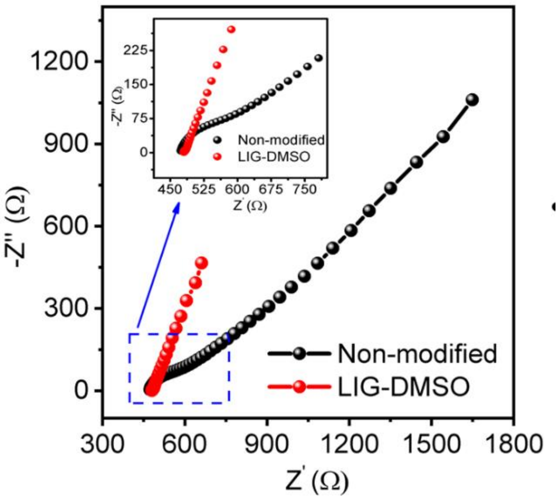

Figure S12. EIS plot obtained in the presence of 1.0 mmol L^{-1} $[Fe(CN)_6]^{3-/4-}$ in 0.1 mol L^{-1} KCl solution, applying a constant potential of +0.22 V *vs*. Ag|AgCl|$KCl_{(sat)}$ in the frequency range from 50,000 Hz to 0.1 Hz

Sulfanilamide detection

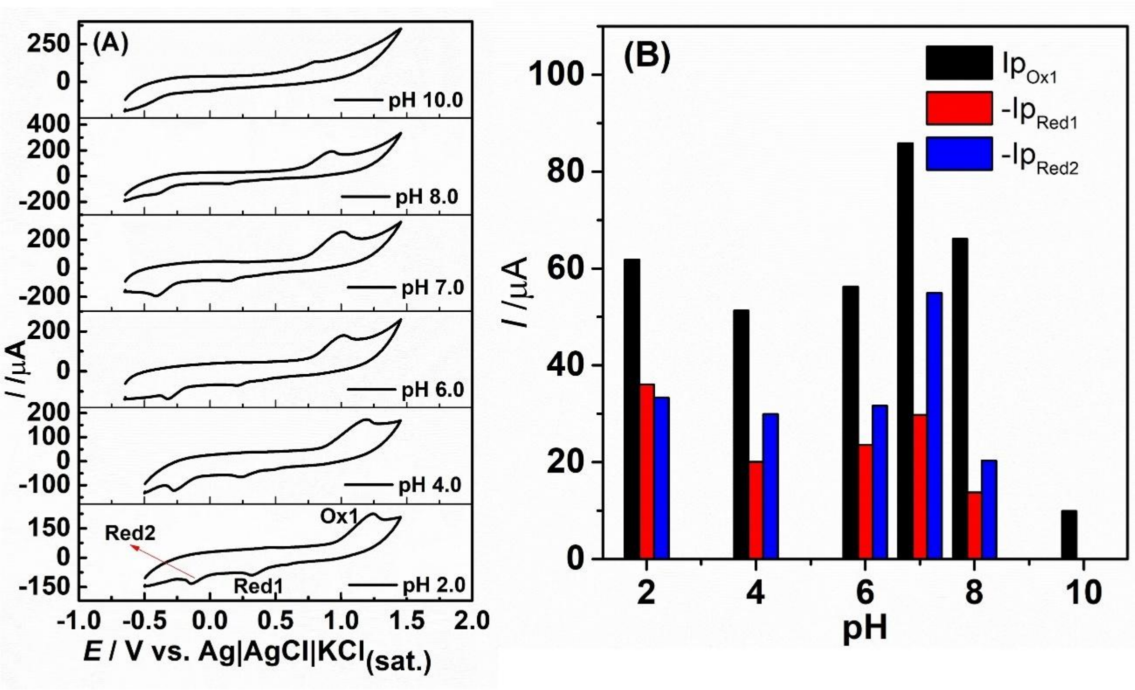

Figure S13. (A) Cyclic voltammograms obtained for SFL (1.0 mmol L^{-1}) in 0.12 mol L^{-1} BR buffer solution (pH range from 2.0 to 12.0). (B) pH influence on peak currents (I_p). Conditions: step potential, 5 mV scan rate, 50 mV s^{-1}.

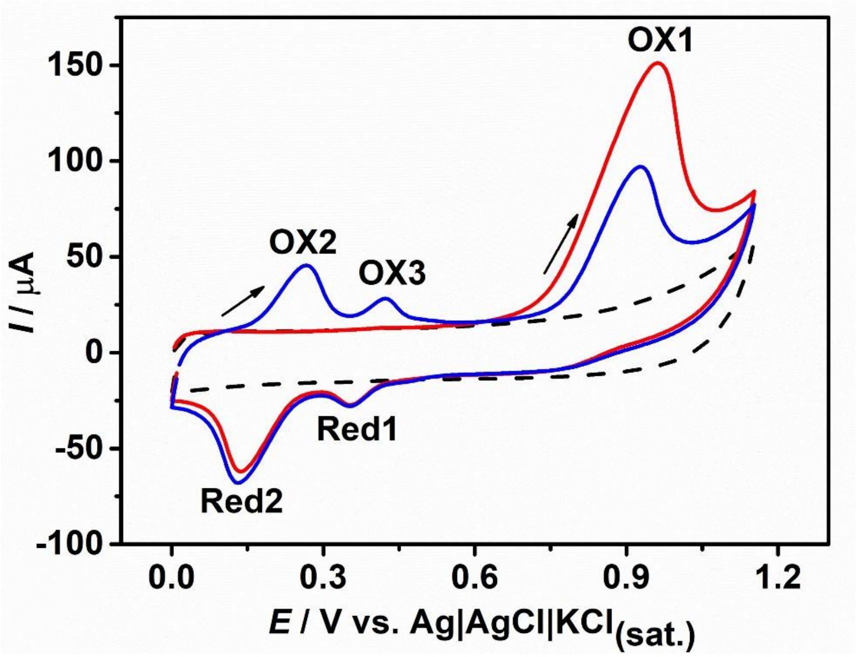

Figure S14. Cyclic voltammetric recordings obtained for 1 mmol L^{-1} SFL (red solid line: first scan; blue solid line: second scan) using DMSO-modified LIG electrode in 0.12 mol L^{-1} BR buffer solution (pH 7.0). Dashed lines correspond to blank; CV conditions: step potential, 5 mV scan rate, 50 mV s^{-1}.

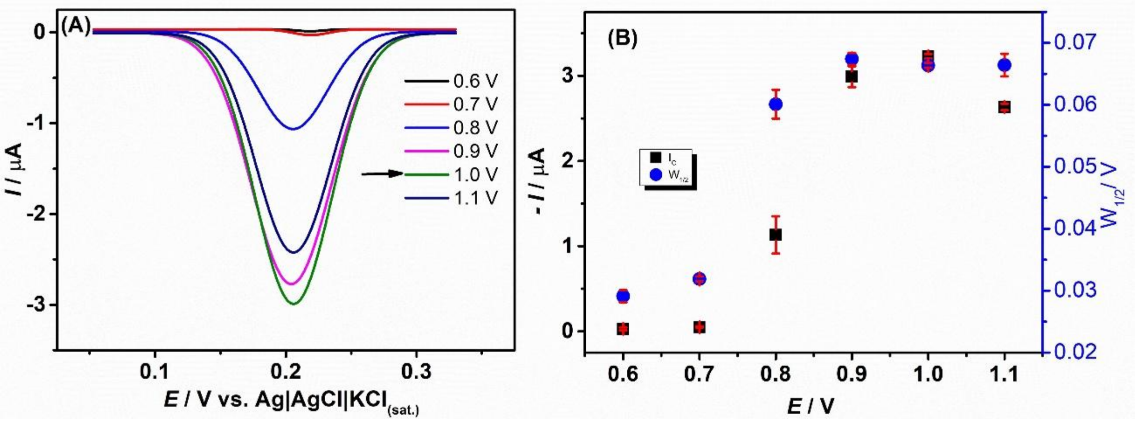

Figure S15. (A) Baseline-corrected DPV voltammograms obtained for 5 µmol L^{-1} of SFL in 0.12 mol L^{-1} BR buffer (pH 7.0) as function of applied potential. (B) Peak currents (black squares) and peak width half height (blue squares) as function of applied potential. Conditions: accumulation time, 20 s; step potential, 5 mV; modulation amplitude, 50 mV; modulation time, 50 ms and interval time, 0.5 s.

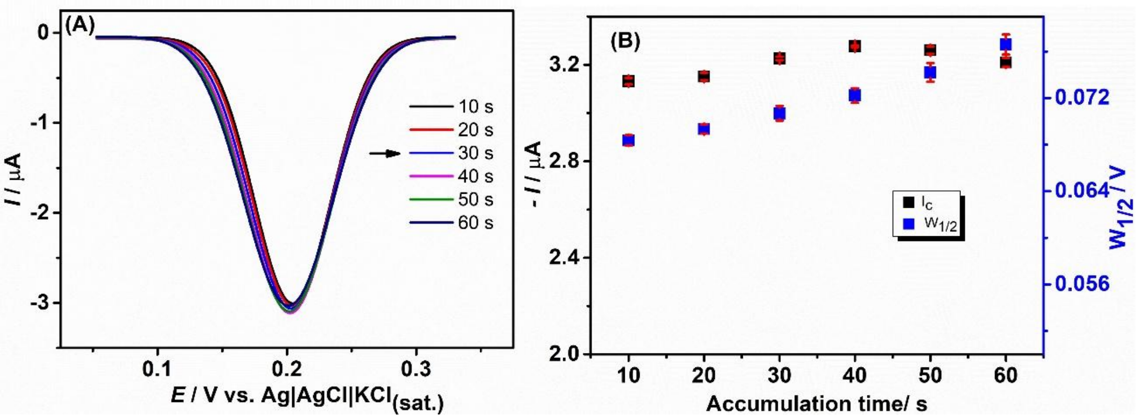

Figure S16. (A) Baseline-corrected DPV responses recorded for 5 µmol L^{-1} of SFL in 0.12 mol L^{-1} BR buffer (pH 7.0) as a function of accumulation time. (B) Peak currents (black squares) and peak width at half height (blue squares) as a function of accumulation time. Conditions: aplied potential, 1.0 V; step potential, 5 mV; modulation amplitude, 50 mV; modulation time, 50 ms and interval time, 0.5 s.

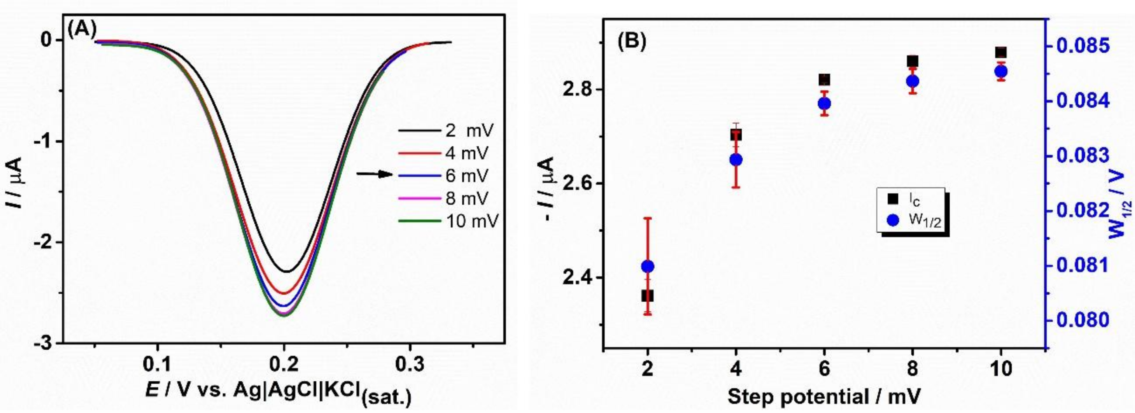

Figure S17. (A) Baseline-corrected DPV voltammograms obtained for 5 µmol L^{-1} of SFL in 0.12 mol L^{-1} BR buffer (pH 7.0) as a function of step potential. (B) Peak currents (black squares) and peak width half height (blue squares) as a function of step potential. Conditions: applied potential, 1.0 V; accumulation time, 30 s; modulation amplitude, 50 mV; modulation time, 50 ms and interval time, 0.5 s.

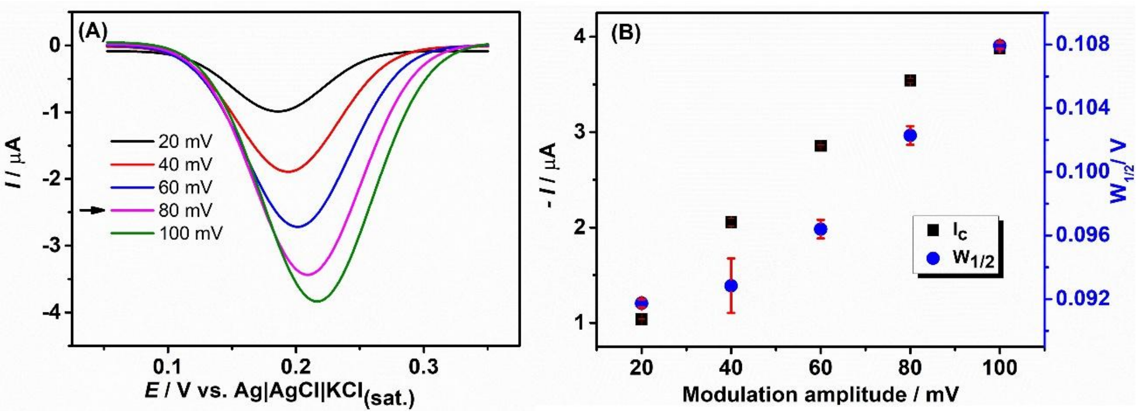

Figure S18. (A) Baseline-corrected DPV voltammograms obtained for 5 µmol L^{-1} of SFL in 0.12 mol L^{-1} BR buffer (pH 7.0) as a function of modulation amplitude. (B) Peak currents (black squares) and peak width half height (blue squares) as a function of modulation amplitude. Conditions: applied potential, 1.0 V; accumulation time, 30 s; step potential, 6mV; modulation time, 50 ms and interval time, 0.5 s.

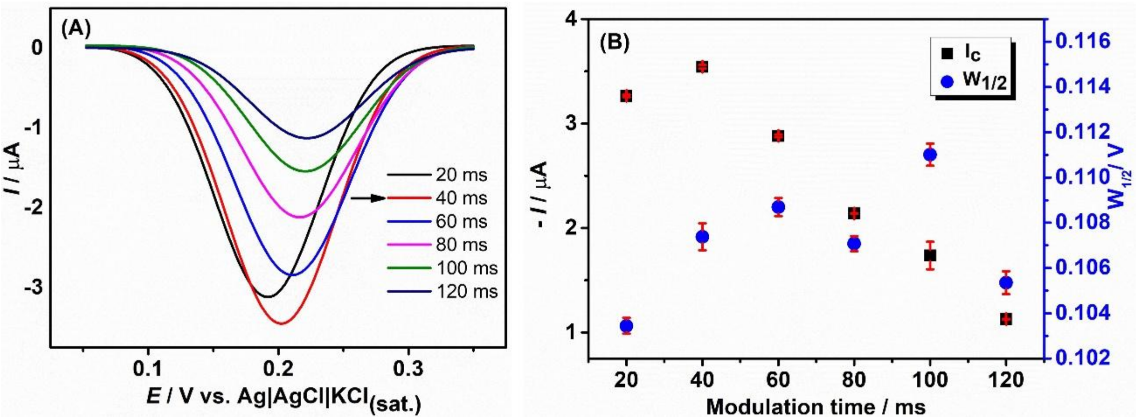

Figure S19. (A) Baseline-corrected DPV voltammograms obtained for 5 µmol L^{-1} of SFL in 0.12 mol L^{-1} BR buffer (pH 7.0) as a function of modulation time, 50 ms. (B) Peak currents (black squares) and peak width half height (blue squares) as a function of modulation time. Conditions: applied potential, 1.0 V; accumulation time, 30 s; step potential, 6 mV; modulation amplitude, 80 mV and interval time, 0.5 s.

Table S2 Optimization of the experimental conditions for the determination of SFL by DPV.

Parameters	Studied interval	Selected condition/value
Supporting electrolyte	BR buffer (pH 2.0-10)	BR (pH 7.0)
Step potential	2–10 mV	6 mV
Modulation amplitude	20 – 100 mV	80 mV
Modulation time	20-120 ms	40 ms
Applied potential	0.6 - 1.1 V	1.0 V
Accumulation time	0 – 60 s	30 s

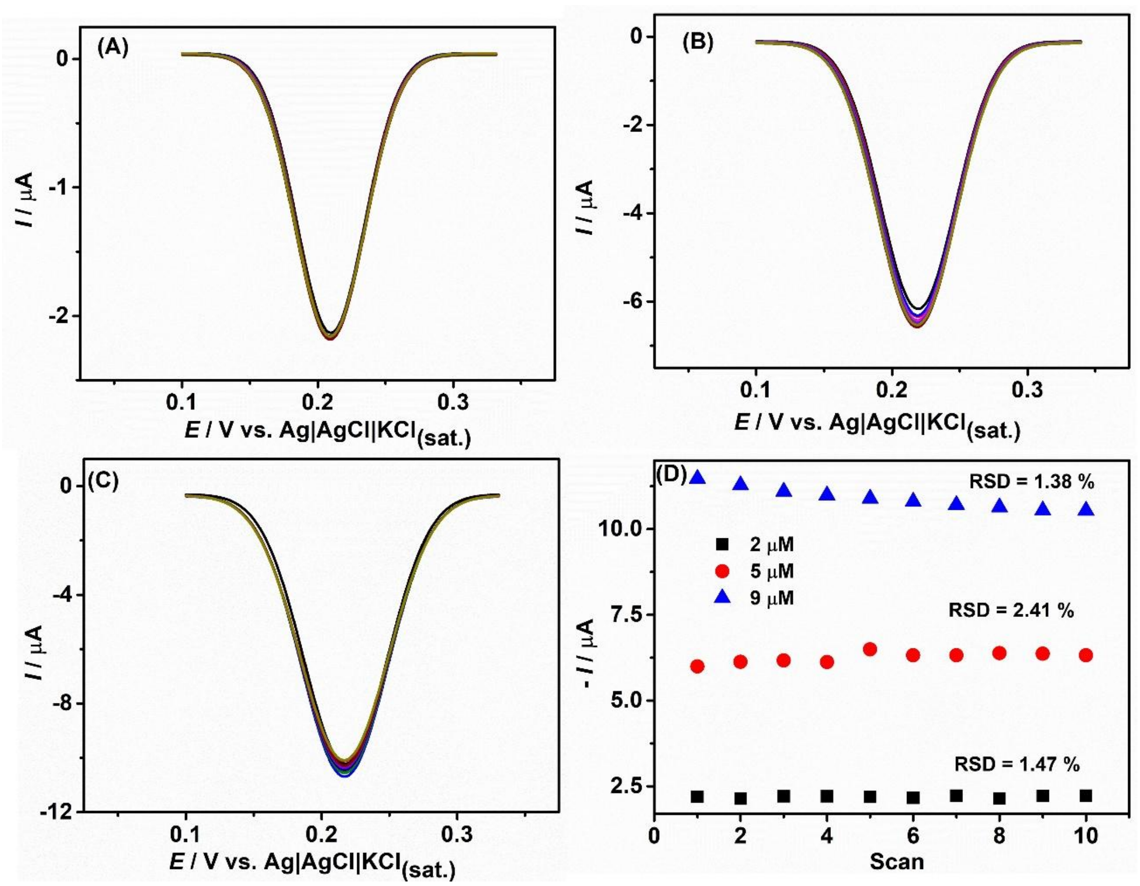

Figure S20. Repeatability data obtained from successive DPV scans (n = 10) for SFL solution containing: (A) 2; (B) 5 and (C) 9 µmol L^{-1}; (D) Peak currents as a function of scan for the experiments in the presence of 2 (squares), 5 (circles) and 9 µmol L^{-1} (triangles). DPV optimized conditions are listed in Table S2.

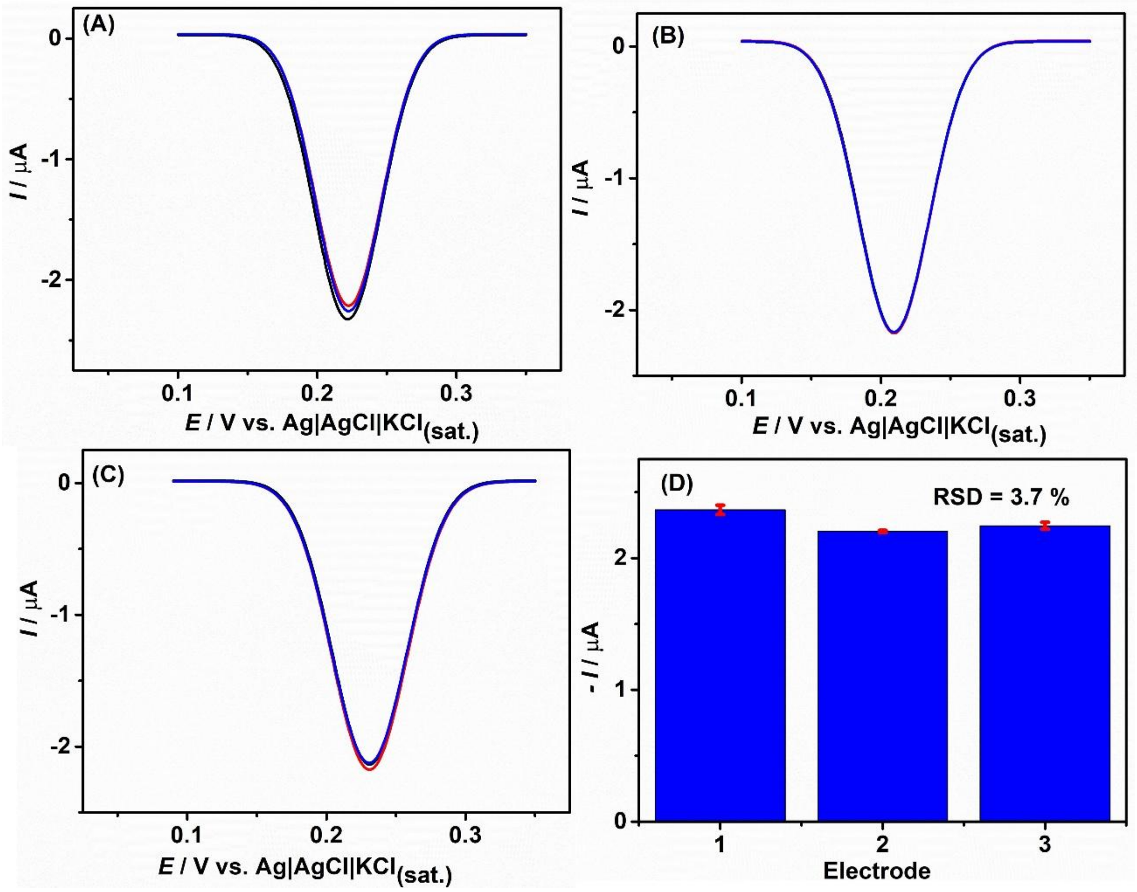

Figure S21. Inter-electrode precision data obtained from successive DPV scans (n = 3) for SFL solution containing 2 µmol L^{-1} at three different electrodes (different batches): (A) electrode #1, (B) electrode #1, and (C) electrode #1. (D) Bar plot showing average current (n=3) for each electrode with the respective error bars (in red). RSD value obtained for the three electrodes. Conditions: see Table S2.

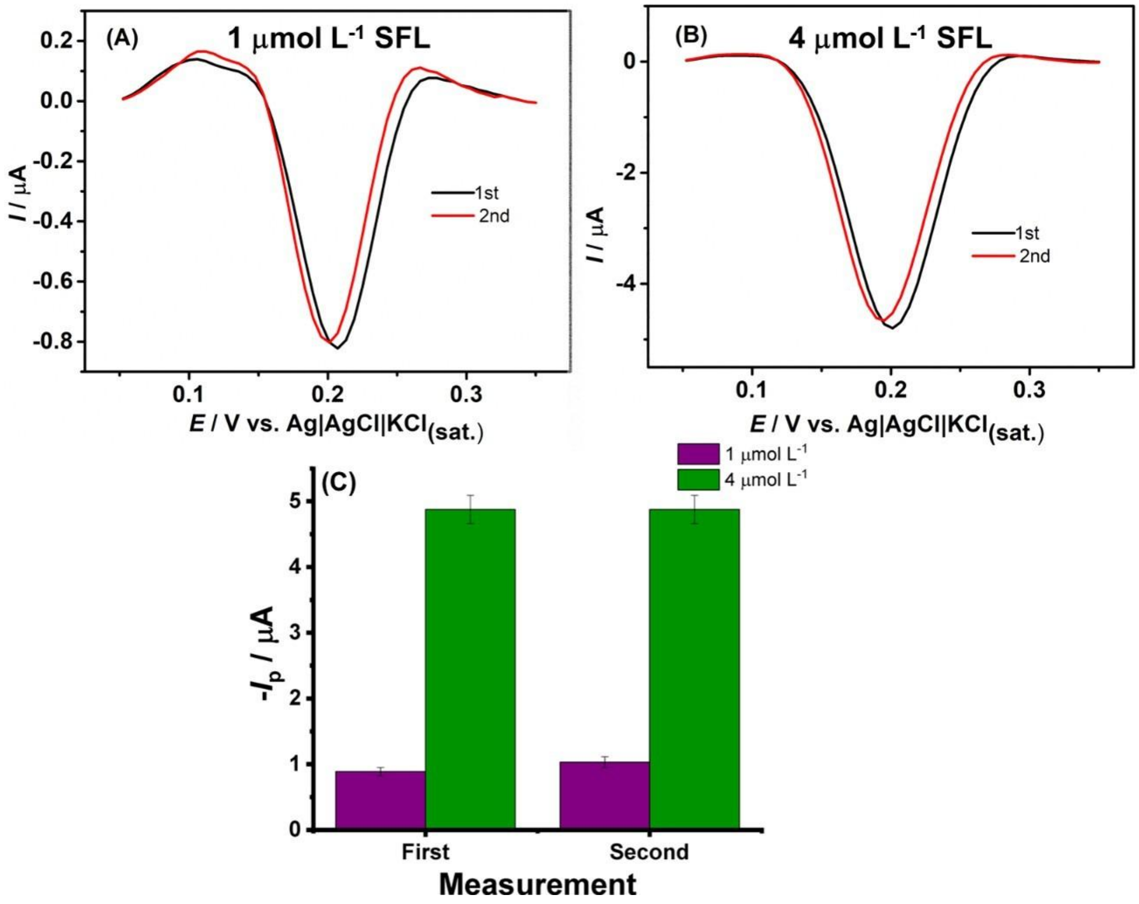

Figure S22 (A) The electrochemical response obtained for the proposed sensor in the presence of (A) 1 μmol L^{-1} and (B) 4 μmol L^{-1} SFL in the first (black line) and second (red line) measurement in order to evaluate the hysteresis of the sensor and (c) respective peak current signal (*I*p). The experiment was assessed by first recording the signal at 1.0 μmol L^{-1} followed by 4.0 μmol L^{-1}, and subsequently in reverse order (4.0 and 1.0 μmol L^{-1}). DPV conditions: see Table S2.

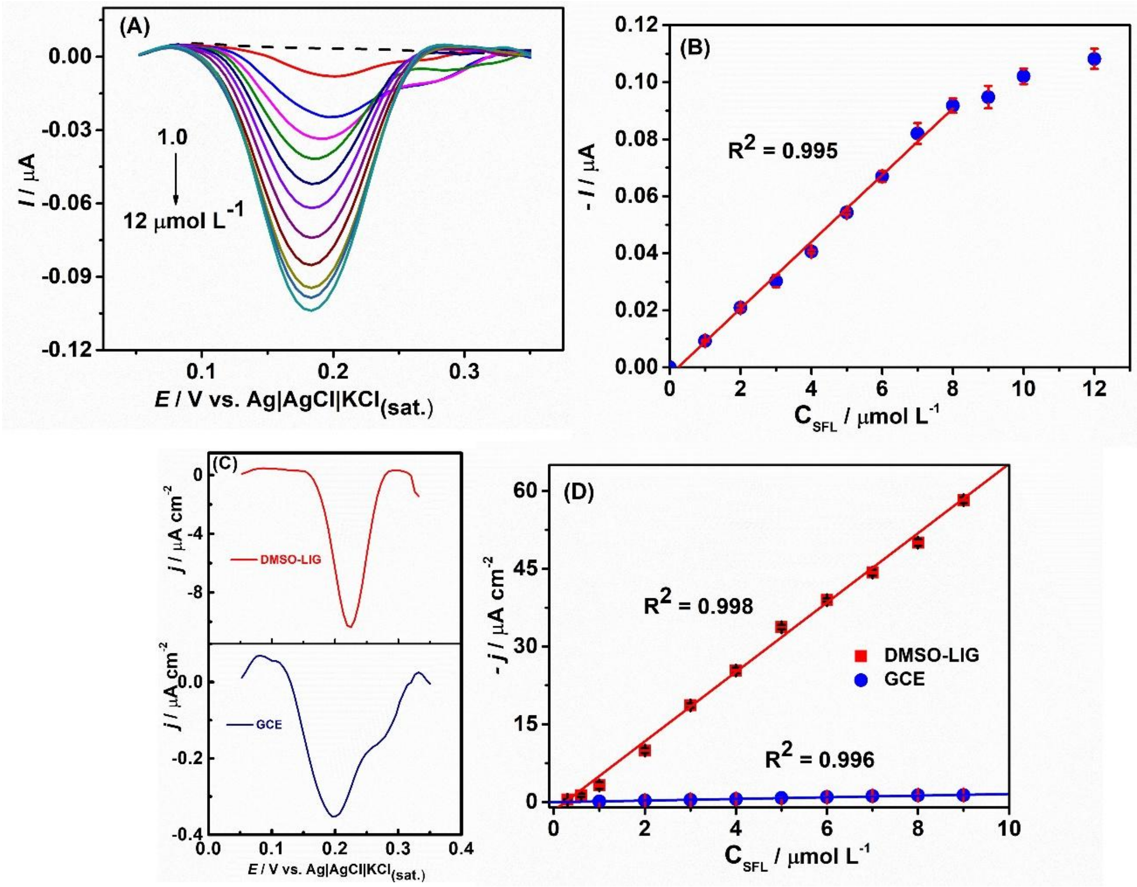

Figure S23. DPV recordings for SFL in BR buffer solution (pH 7.0) on (A) GCE (1 to 12.0 µmol L^{-1}) under optimized experimental conditions are summarized in Table S2, and (B) respective calibration curve; (C) DPV voltammograms for SFL (2 µmol L^{-1}) utilizing DMSO-treated LIG electrode (solid red line) and GCE (blue solid line); (D) Relationship between current density and SFL concentration obtained utilizing both electrodes.

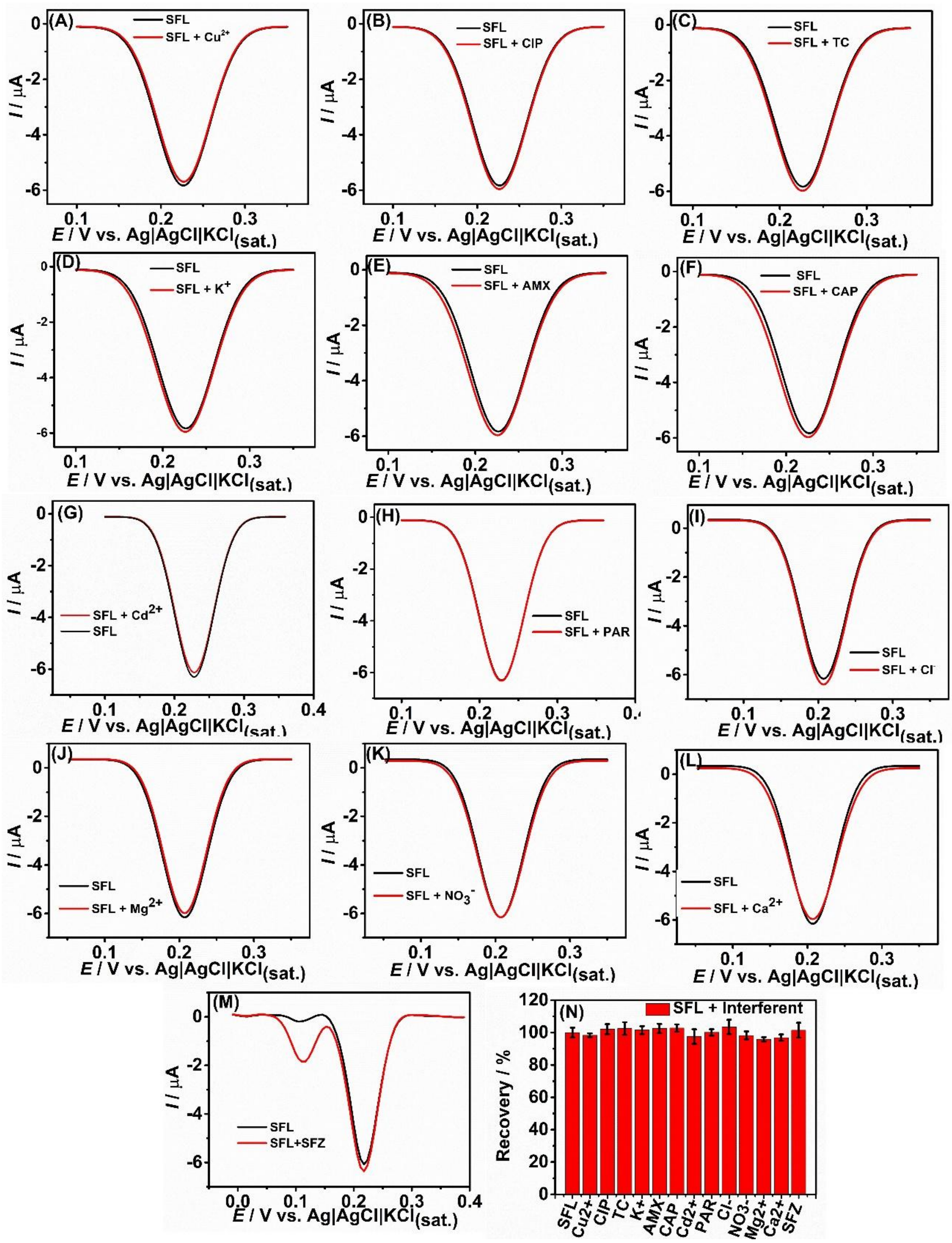

Figure S24. Baseline-corrected DPV voltammograms obtained for 5 μmol L^{-1}SFL and the equimolar mixture (5.0 μmol L^{-1}) of SFL and interferents (A) Cu^{2+}; (B) CIP; (C) TC; (D) K^{+}; (E) AMX; (F) CAP; (G) Cd^{2+}; (H) PAR; (I) Cl^{-}; (J) Mg^{2+}; (K) NO_3^{-}; (L) Ca^{2+}; (M) SFZ under optimized instrumental DPV conditions (Table S2); (N) Effect of concomitant interfering species on the electrochemical response of SFL (1:1 concentration ratio of SFL and interfering species).

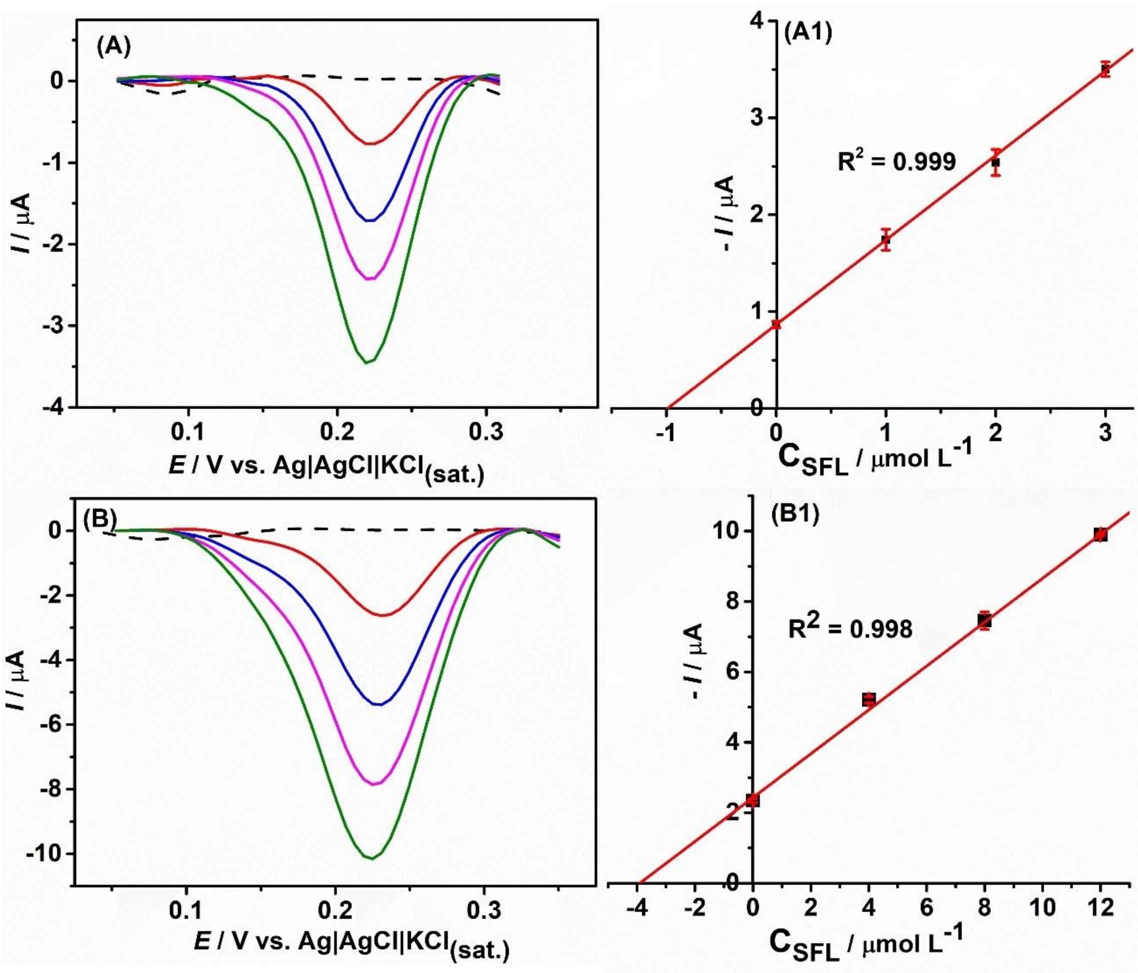

Figure S25. Baseline-corrected DPV responses (n = 3) for the detection of SFL in tap water samples spiked with (A) 1 µmol L^{-1} and (B) 4 µmol L^{-1} SFL, followed by three successive additions of standard SFL solutions. The corresponding calibration curves are shown adjacent to each set of DPV scans. In all plots, the first scan corresponds to the blank, the second to the spiked sample; and the third, fourth, and fifth scans correspond to successive additions of the SFL standard solution. No sample dilution required. Optimized conditions are listed in Table S2.

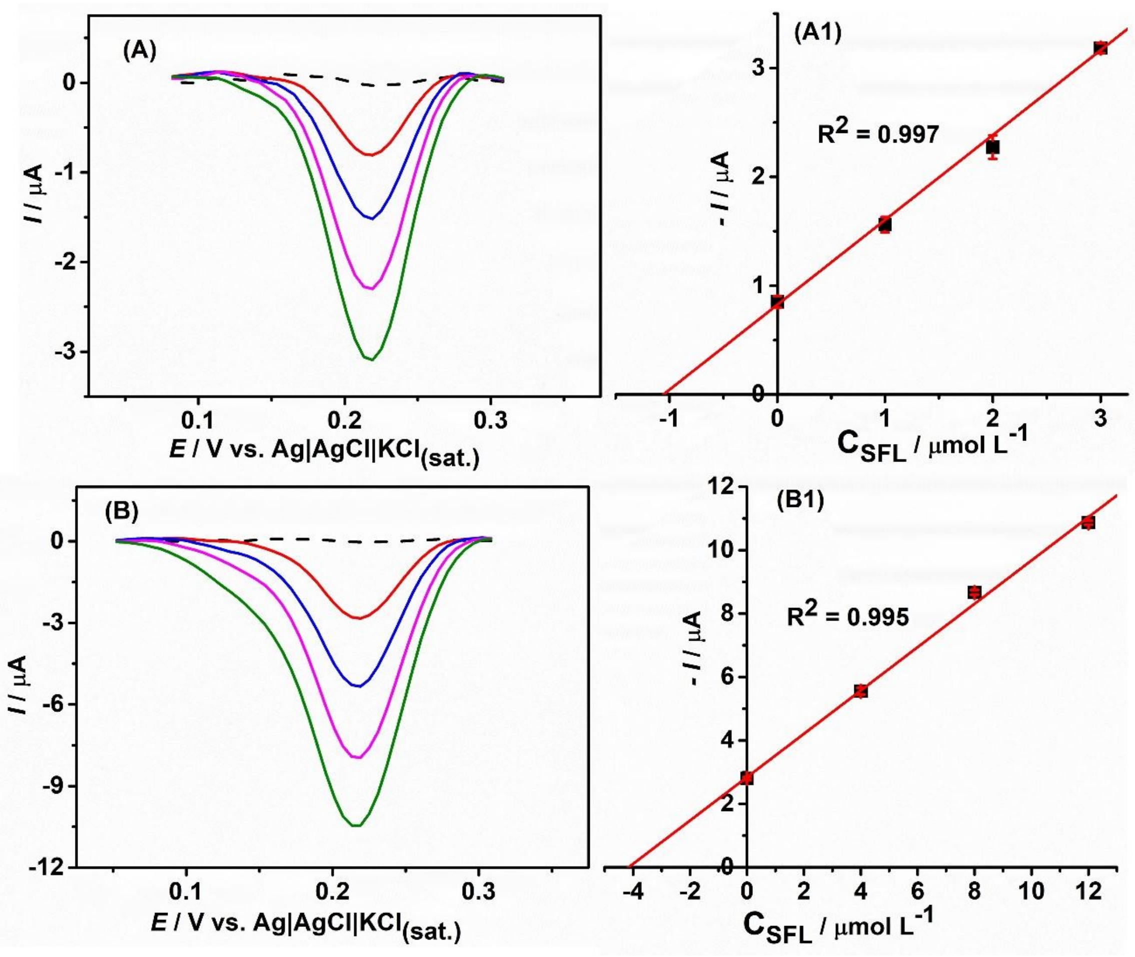

Figure S26. Baseline-corrected DPV responses (n = 3) for the detection of SFL in drinking water samples spiked with (A) 1 µmol L^{-1} and (B) 4 µmol L^{-1}, followed by three successive additions of standard SFL solutions. The respective calibration curves are shown beside each set of DPV scans. In all plots, the first scan corresponds to the blank, the second to the spiked sample; and the third, fourth, and fifth scans correspond to successive additions of the SFL standard solution. No sample dilution required. Optimized conditions are listed in Table S2.

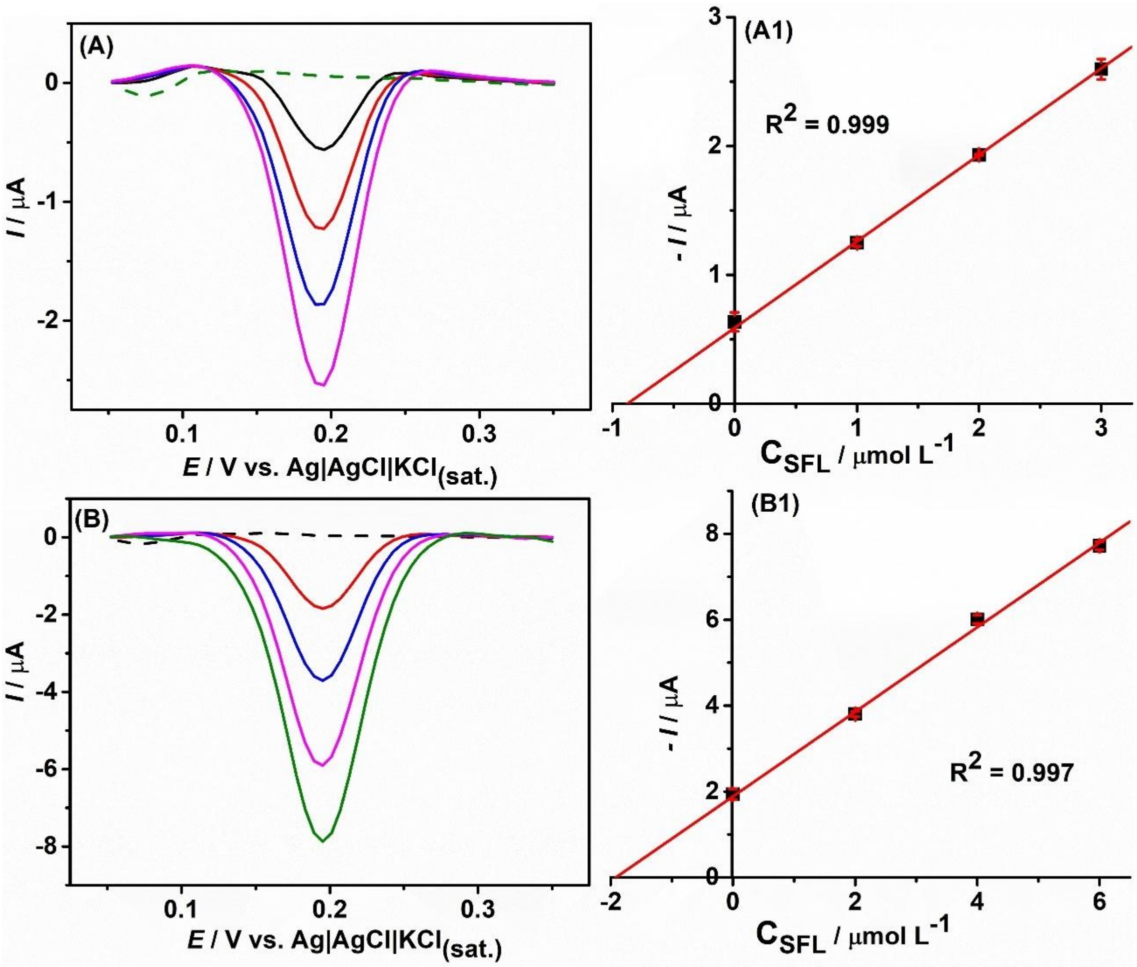

Figure S27. Baseline-corrected DPV responses (n = 3) for the detection of SFL in synthetic urine samples spiked with (A) 1 µmol L^{-1} and (B) 2 µmol L^{-1} SFL, followed by three successive additions of standard SFL solutions. The corresponding calibration curves are shown adjacent to each set of DPV scans. In all plots, the first scan corresponds to the blank, the second to the spiked sample; and the third, fourth, and fifth scans correspond to successive additions of the SFL standard solution. The sample was 10-fold diluted in supporting electrolyte. Optimized conditions are listed in Table S2.

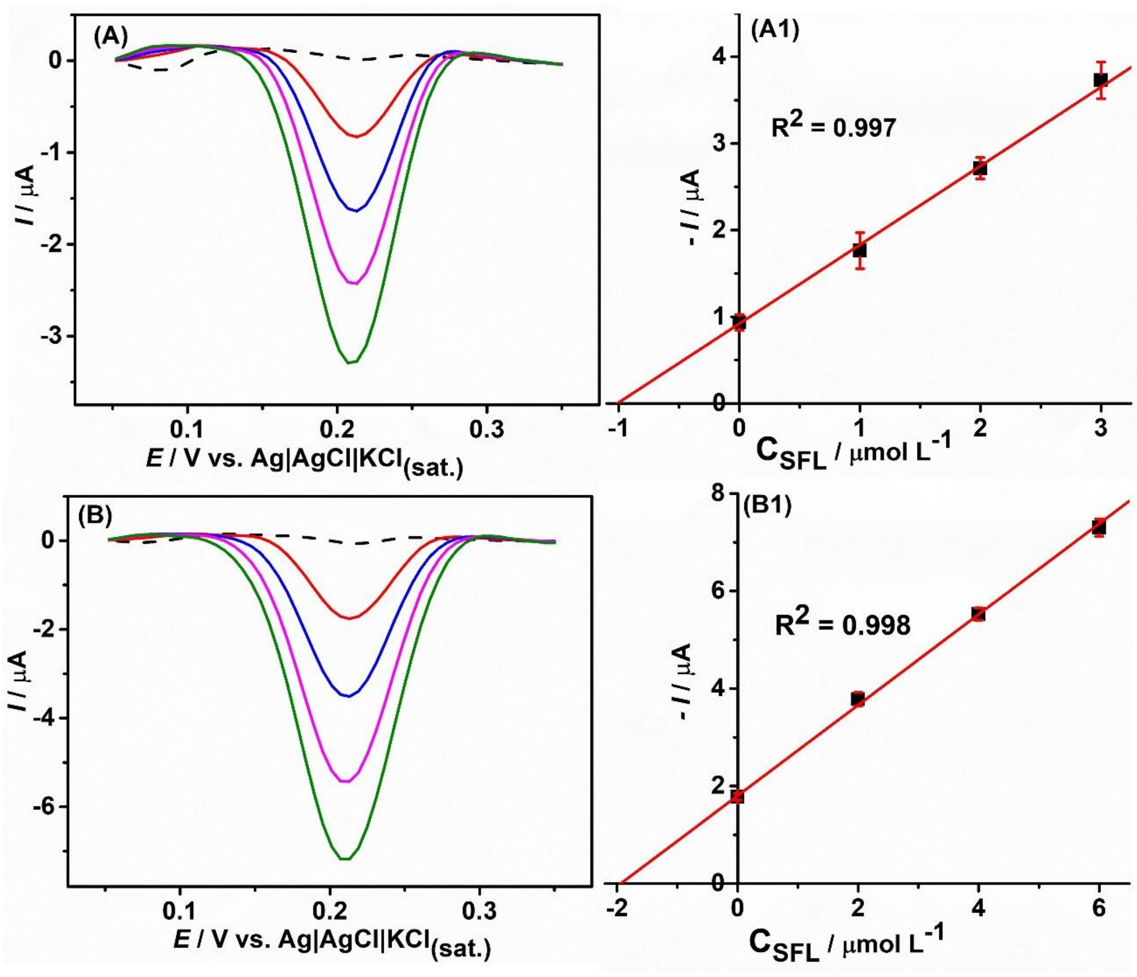

Figure S28. Baseline-corrected DPV responses (n = 3) for the detection of SFL in river water samples spiked with (A) 1 µmol L^{-1} and (B) 2 µmol L^{-1} SFL, followed by three successive additions of standard SFL solutions. The corresponding calibration curves are shown adjacent to each set of DPV scans. In all plots, the first scan corresponds to the blank, the second to the spiked sample; and the third, fourth, and fifth scans correspond to successive additions of the SFL standard solution. The sample was diluted 4 times in supporting electrolyte. Optimized conditions are listed in Table S2.

Table S3 Comparison of the proposed method with other eletroanalytical methods reported in literature for SFL sensing

Electrode[1]	Method[2]	LOD/ µmol L^{-1}	Linear Range/ µmol L^{-1}	Sensitivity/ µA Lµmol^{-1}	Samples	Reference
LSG -MWCNT	SWV<br>BIA-AD	1.30<br>2.33	10.0 -115.0<br>10.0-150.0	0.67	urine, lake water, tap water, mineral water, and drug (otologic solution)	6
Ag@Pt-Rh NCs/ GCE	DPV	0.26	2.6 –320.0	0.02	Groundwater, yellow river and tap water	7
rGO / GCE	BIA-AD	2.30	10.0 –50.0	0.59	Lake water and synthetic saliva, sweat and urine	8
3D printed CB-PLA	SWV	0.26	1.0-10.0	1.69	Honey	9
MIP/GO/GCE	SWV	n.m	0.06 - 0.6<br>1.16 - 5.80	25.83<br>1.45	Standard aqueous solution	10
3D printed CB/PLA	SWV	0.20	2.0 – 11.0	0.26	Wastewater and tap water	11
carboxyl/DMF/MWCNTs/GCE	CV	0.50	1.0- 100	0.035	Pork	12
CO_2-GS	SWV	0.11	1.0 -90	0.04	Water and urine	13
MIP/PGE	DPV	0.02	0.05 -1.10<br>1.1 - 48	1.16<br>0.01	Blood serum and ground water	14
3D printed CB/PLA	DPV	0.012	1.0 - 39.2		Breast milk, synthetic urine, and pharmaceutical formulation	15
PBCE	DPV	4.1	10.0 -100.0	0.076	Milk, synthetic urine, otologic solution, lake and seawater	16
GCE	SWV	0.92	5.0-74.7	0.067	Otologic solution, human urine and human serum	17

LIG -DMSO	DPV	0.09	0.3 - 9	1.27	Synthetic urine, Tap, drinking and river water	This work

SWV: square wave voltammetry; BIA-AD – batch injection analysis with amperometric detection; DPV – differential pulse voltammetry; CV – cyclic voltammetry.

LSG -MWCNT – laser-scribed graphene electrode (obtained from the phenolic resin substrate) modified with multi-walled carbon Nanotube; MIP/GO/GCE - molecularly imprinted polymer combined with graphite oxide modified on glassy carbon electrode; CO_2-GS - CO_2-plasma-treated graphite sheets electrode; MIP/PGE - Molecularly imprinted polymers modified pencil graphite electrode; Ag@Pt-Rh NCs/ GCE- glassy carbon electrode modified with trimetallic Ag@Pt-Rh core–shell NCs nanocubes; rGO/ GCE– reduced graphene oxide modified glassy carbon electrode; CB/PLA – 3D printed carbon black integrated polylactic acid electrode; carboxyl/DMF/MWCNTs/GCE - Carboxyl multiwalled carbon nanotubes through ultrasonic dispersing in dimethylfomamide Modified glassy carbon electrode; n.m – not mentioned; PBCE- Paper-based carbon electrode; LIG - DMSO – Dimethyl sulfoxide-modified laser-induced graphene electrode.

Supporting information for:

Table S4. Comparison between the analytical features obtained using glassy carbon electrode (GCE) and DMSO-LIG, using DPV technique, and optimized conditions described in Table S2. These values were normalized using the geometric area of each electrode.

Analytical features	DMSO-LIG	GCE
**Linear range (µmol L^{-1})**	0.3 – 9.0	1.0 – 8.0
**Sensitivity (µA cm^{-2} L $µmol^{-1}$)**	6.7 ± 0.1	0.160 ± 0.003
**Intercept / (µA)**	-1.7 ± 0.3	-0.04 ± 0.01
**R^2**	0.998	0.996

Table S5. Results of spiked-recovery experiments and statistical comparison (*t* and *F* values at 95% confidence level) obtained for the proposed electrochemical method and the spectrophotometric reference method in tap, drinking and river water and synthetic urine samples (n =3).

	Proposed method			Spectrophotometric method			t_{crit}	F_{crit}
**Sample**	**Spiked /µmol L^{-1}**	**Found ± SD / µmol L^{-1}**	**Recovery ± SD / %**	**Spiked /µmol L^{-1}**	**Found ± SD / µmol L^{-1}**	**Recovery ± SD / %**		
**Tap water**	4.0	3.9 ± 0.1	98± 3	4.0	3.86 ± 0.16	97± 4	0.367	2.56
**Drinking water**	4.0	4.1 ± 0.1	103 ± 3	4.0	3.98 ± 0.06	100 ± 2	1.78	2.78
**River water**	8.0	7.6 ± 0.4	95 ± 1	8.0	8.04 ± 0.32	101 ± 4	1.49	1.56
**Synthetic urine**	20.0	19 ± 1	95 ± 5	20.0	18.5±0.3	93±2	2.776	11.11

$t_{theorical}$ = 2.776 and $F_{theorical}$. = 19

Supporting information for:

Reference

(1) Brooks, T.; Keevil, C. W. A Simple Artificial Urine for the Growth of Urinary Pathogens. *Letters in Applied Microbiology* **1997**, *24* (3), 203–206. https://doi.org/10.1046/j.1472-765X.1997.00378.x.

(2) Greczynski, G.; Hultman, L. The Same Chemical State of Carbon Gives Rise to Two Peaks in X-Ray Photoelectron Spectroscopy. *Sci Rep* **2021**, *11* (1), 11195. https://doi.org/10.1038/s41598-021-90780-9.

(3) Cardoso, R. M.; Mendonça, D. M. H.; Silva, W. P.; Silva, M. N. T.; Nossol, E.; da Silva, R. A. B.; Richter, E. M.; Muñoz, R. A. A. 3D Printing for Electroanalysis: From Multiuse Electrochemical Cells to Sensors. *Anal Chim Acta* **2018**, *1033*, 49–57. https://doi.org/10.1016/j.aca.2018.06.021.

(4) Voiry, D.; Chhowalla, M.; Gogotsi, Y.; Kotov, N. A.; Li, Y.; Penner, R. M.; Schaak, R. E.; Weiss, P. S. Best Practices for Reporting Electrocatalytic Performance of Nanomaterials. *ACS Nano* **2018**, *12* (10), 9635–9638. https://doi.org/10.1021/acsnano.8b07700.

(5) Nagaraja, P.; Naik, S.; Shrestha, A.; Shivakumar, A. A Sensitive Spectrophotometric Method for the Determination of Sulfonamides in Pharmaceutical Preparations. *Acta Pharmaceutica* **2007**, *57* (3), 333–42. https://doi.org/10.2478/v10007-007-0026-4.

(6) M. de Farias, D.; Pradela-Filho, L. A.; Arantes, I. V. S.; Gongoni, J. L. M.; Veloso, W. B.; Meloni, G. N.; Paixão, T. R. L. C. Sulfanilamide Electrochemical Sensor Using Phenolic Substrates and CO 2 Laser Pyrolysis. *ACS Appl Mater Interfaces* **2023**, *15* (48), 56424–56432. https://doi.org/10.1021/acsami.3c11462.

(7) Zhang, Y.; Lv, Y.; Chen, Y.; Li, Y.; Wang, Y.; Zhao, H. Trimetallic Ag@Pt-Rh Core-Shell Nanocubes Modified Anode for Voltammetric Sensing of Dopamine and Sulfanilamide. *Chemical Engineering Science* **2022**, *249*, 117326. https://doi.org/10.1016/j.ces.2021.117326.

(8) de Faria, L. V.; Lisboa, T. P.; Matias, T. A.; de Sousa, R. A.; Matos, M. A. C.; Munoz, R. A. A.; Matos, R. C. Use of Reduced Graphene Oxide for Sensitive Determination of Sulfanilamide in Synthetic Biological Fluids and Environmental Samples by Batch Injection Analysis. *Journal of Electroanalytical Chemistry* **2021**, *892*, 115298. https://doi.org/10.1016/j.jelechem.2021.115298.

(9) Rocha, R. G.; de Faria, L. V.; Silva, V. F.; Muñoz, R. A. A.; Richter, E. M. Carbon Black Integrated Polylactic Acid Electrodes Obtained by Fused Deposition Modeling: A Powerful Tool for Sensing of Sulfanilamide Residues in Honey Samples. *Journal of Agricultural and Food Chemistry* **2023**, *71* (6), 3060–3067. https://doi.org/10.1021/acs.jafc.2c07814.

(10) Wei, X.; Xu, X.; Qi, W.; Wu, Y.; Wang, L. Molecularly Imprinted Polymer/Graphene Oxide Modified Glassy Carbon Electrode for Selective

Detection of Sulfanilamide. *Progress in Natural Science: Materials International* **2017**, *27* (3), 374–379. https://doi.org/10.1016/j.pnsc.2017.05.001.

(11) Di-Oliveira, M.; Araújo, D. A. G.; Ramos, D. L. O.; Faria, L. V. de; Rocha, R. G.; Sousa, R. M. F.; Richter, E. M.; Paixão, T. R. L. C.; Munoz, R. A. A. Sequential Cyclic-Square-Wave Voltammetric Determination of Sulfanilamide and Ciprofloxacin in Environment Water Samples Using a 3D-Printed Electrochemical Device. *Electrochim Acta* **2024**, *481*, 143945. https://doi.org/10.1016/j.electacta.2024.143945.

(12) He, B.; Chen, W. Carboxyl Multiwalled Carbon Nanotubes through Ultrasonic Dispersing in Dimethylfomamide Modified Electrode as a Sensitive Amperometric Sensor for Detection of Sulfonamide. *International Journal of Electrochemical Science* **2015**, *10* (5), 4335–4345. https://doi.org/10.1016/S1452-3981(23)06626-9.

(13) Pereira, J. F. S.; Di-Oliveira, M.; Faria, L. V.; Borges, P. H. S.; Nossol, E.; Gelamo, R. V.; Richter, E. M.; Lopes, O. F.; Muñoz, R. A. A. CO2-Plasma Surface Treatment of Graphite Sheet Electrodes for Detection of Chloramphenicol, Ciprofloxacin and Sulphanilamide. *Microchimica Acta* **2023**, *190* (10), 379. https://doi.org/10.1007/s00604-023-05953-2.

(14) Tadi, K. K.; Motghare, R. V.; Ganesh, V. Electrochemical Detection of Sulfanilamide Using Pencil Graphite Electrode Based on Molecular Imprinting Technology. *Electroanalysis* **2014**, *26* (11), 2328–2336. https://doi.org/10.1002/elan.201400251.

(15) Lisboa, T. P.; Alves, G. F.; de Faria, L. V.; de Souza, C. C.; Matos, M. A. C.; Matos, R. C. 3D-Printed Electrode an Affordable Sensor for Sulfanilamide Monitoring in Breast Milk, Synthetic Urine, and Pharmaceutical Formulation Samples. *Talanta* **2022**, *247*, 123610. https://doi.org/10.1016/j.talanta.2022.123610.

(16) Lisboa, T. P.; de Faria, L. V.; Alves, G. F.; Matos, M. A. C.; Matos, R. C. Development of Paper Devices with Conductive Inks for Sulfanilamide Electrochemical Determination in Milk, Synthetic Urine, and Environmental and Pharmaceutical Samples. *Journal of Solid State Electrochemistry* **2021**, *25* (8–9), 2301–2308. https://doi.org/10.1007/s10008-021-05002-z.

(17) Ferraz, B. R. L.; Guimarães, T.; Profeti, D.; Profeti, L. P. R. Electrooxidation of Sulfanilamide and Its Voltammetric Determination in Pharmaceutical Formulation, Human Urine and Serum on Glassy Carbon Electrode. *Journal of Pharmaceutical Analysis* **2018**, *8* (1), 55–59. https://doi.org/10.1016/j.jpha.2017.10.004.
